# Human skeletal muscle tissue chip autonomous payload reveals changes in fiber type and metabolic gene expression due to spaceflight

**DOI:** 10.1038/s41526-023-00322-y

**Published:** 2023-09-15

**Authors:** Maddalena Parafati, Shelby Giza, Tushar S. Shenoy, Jorge A. Mojica-Santiago, Meghan Hopf, Legrand K. Malany, Don Platt, Isabel Moore, Zachary A. Jacobs, Paul Kuehl, Jason Rexroat, Gentry Barnett, Christine E. Schmidt, William T. McLamb, Twyman Clements, Paul M. Coen, Siobhan Malany

**Affiliations:** 1 Department of Pharmacodynamics, College of Pharmacy, University of Florida, Gainesville, FL 32610 USA; 2 J. Crayton Pruitt Family Department of Biomedical Engineering, Herbert Wertheim College of Engineering, University of Florida, Gainesville, FL 32610 USA; 3 Translational Research Institute, AdventHealth, Orlando, FL 32804 USA; 4 Micro-gRx, INC, Orlando, FL 32935 USA; 5 Micro Aerospace Solutions, INC, Melbourne, FL 32935 USA; 6 Space Tango, LLC, Lexington, KY 40505 USA

**Keywords:** Lab-on-a-chip, Genetic databases

## Abstract

Microphysiological systems provide the opportunity to model accelerated changes at the human tissue level in the extreme space environment. Spaceflight-induced muscle atrophy experienced by astronauts shares similar physiological changes to muscle wasting in older adults, known as sarcopenia. These shared attributes provide a rationale for investigating molecular changes in muscle cells exposed to spaceflight that may mimic the underlying pathophysiology of sarcopenia. We report the results from three-dimensional myobundles derived from muscle biopsies from young and older adults, integrated into an autonomous CubeLab™, and flown to the International Space Station (ISS) aboard SpaceX CRS-21 as part of the NIH/NASA funded Tissue Chips in Space program. Global transcriptomic RNA-Seq analyses comparing the myobundles in space and on the ground revealed downregulation of shared transcripts related to myoblast proliferation and muscle differentiation. The analyses also revealed downregulated differentially expressed gene pathways related to muscle metabolism unique to myobundles derived from the older cohort exposed to the space environment compared to ground controls. Gene classes related to inflammatory pathways were downregulated in flight samples cultured from the younger cohort compared to ground controls. Our muscle tissue chip platform provides an approach to studying the cell autonomous effects of spaceflight on muscle cell biology that may not be appreciated on the whole organ or organism level and sets the stage for continued data collection from muscle tissue chip experimentation in microgravity. We also report on the challenges and opportunities for conducting autonomous tissue-on-chip CubeLab^TM^ payloads on the ISS.

## Introduction

The number of National Aeronautics and Space Administration (NASA) sponsored payloads to the International Space Station (ISS) National Lab increased nearly 300% between 2010–2020 according to the Center for the Advancement of Science in Space (CASIS) annual reports. The growth, predominately in the areas of technology development, technology demonstration, and life science research, was due to the NASA commercial resupply program, increased financial support for space-based research, and availability of implementation companies offering a diversity of custom payload hardware. The Tissue Chips in Space initiative, a cooperation between the National Institutes of Health (NIH) and NASA has leveraged increased access to the ISS with advanced tissue engineering and microfabrication approaches to investigate microgravity-induced changes on various tissue functions, including those associated with cardiac dysfunction, decreased bone density, muscle wasting and immunosuppression^1–5^. Microphysiological systems (MPS) hold promise to translate discoveries faster to the clinic and increase efficiency and decrease costs, particularly for diseases where animal models do not fully recapitulate human disease pathology and toxicity^6–11^. With the FDA modernization Act 2.0, which allows alternatives to animal testing, MPS are likely to have an even greater impact^12^. The in-space versions promise to be disruptive in modeling age-related diseases. The long-term goals of the Tissue Chips in Space initiative are to uncover molecular mechanisms associated with disease progression related to aging, to advance lab-on-chip technologies for the evaluation of therapeutics to counteract pathologies in vitro with clinically relevant physiology, and to maintain prolonged tissue cultures to study microgravity and radiation effects on chronic human disease progression.

**Fig. 1:**
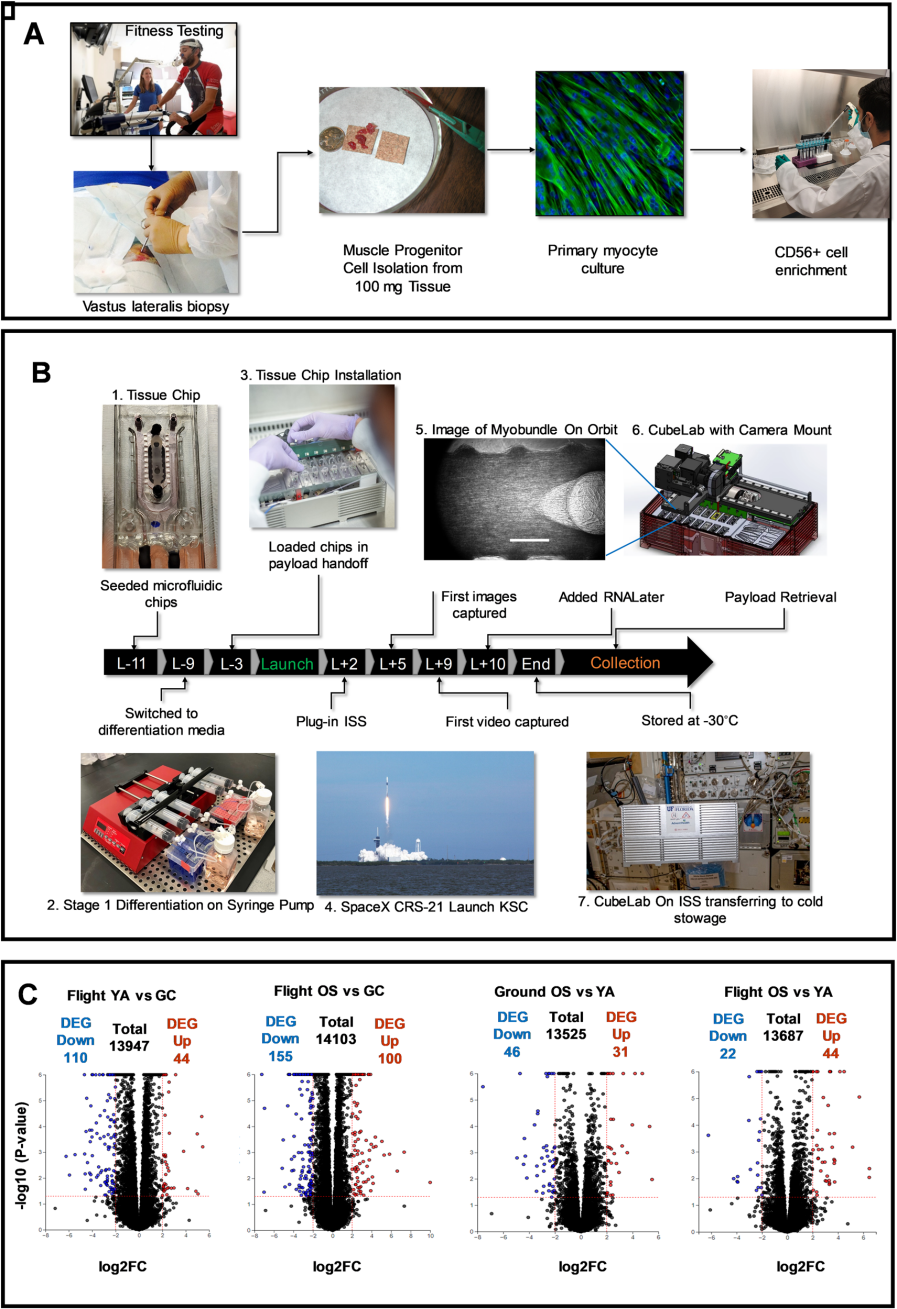
Experimental design, implementation, and RNA analysis of the skeletal muscle MPS payload for the international space station. **A** Human cell banks. Skeletal muscle biopsies were obtained from the vastus lateralis from volunteers (AdventHealth, Orlando). Isolated muscle precursor cultures were enriched for CD56+ (myogenic) cells. **B** Experimental flight timeline. Photos are numbered in the order of the process. 1. Tissue chips were seeded with myoblasts, 2. pre-differentiated using a syringe pump, 3. loaded into the CubeLab™ and 4. launched to the ISS on SpaceX CRS-21. Crew members installed the PAUL on the EXPRESS rack locker and the on-orbit experiment was initiated after plug-in. 5. Images of the myobundles were captured on orbit by the optics system installed into the lid of the CubeLab^TM^. Scalebar = 500 μm. 6. The Optics system automatically moved in the x,y,z axes to collect images. 7. Ten days post launch, crew members moved the payload to cold stowage following experiment termination with RNALater. **C** RNA-Seq volcano plots. YA flight vs ground; OS flight vs ground; Ground OS vs YA and Flight OS vs YA. Colored points are differentially expressed between comparisons. Blue: significantly down-regulated genes. Red: significantly up-regulated genes. Significance is determined according to log2 fold change with threshold set at ±2 and -log10 *p*-value ≤ 0.05. Vertical dotted lines are positioned at log2 fold-change of ±2 and horizontal dotted lines are positioned at the -log10 *p*-value = 0.05. RNA-Seq analysis measured three distinct chips each containing one myobundle derived from five male donor cells pooled in equal ratios.

Our team at the University of Florida has developed a skeletal muscle MPS that, through multiple flights to the ISS, seeks to correlate changes in global transcriptomics and muscle biomechanics with accelerated muscle dysfunction induced by spaceflight^13^. In an increasing aging population, progressive muscle atrophy, or sarcopenia, leads to a dramatic decline in mobility^14^, and yet remains without effective therapeutic options^15,16^. Astronauts, on the other hand, experience significant muscle loss in an accelerated timescale, 20% over two weeks, compared to 1–2% per year for the typical person over age 35^17,18^, such that complete prevention is not attainable with rigorous exercise^19^. In collaboration with AdventHealth, Translational Research Institute (TRI), we obtained percutaneous biopsies of the vastus lateralis from five young, athletic male (<40 yrs, YA) and five older, sedentary male (>60 yrs, OS) volunteers. A participant was considered athletic or sedentary based on their level of routine endurance exercise (running, cycling, or swimming) of at least 3 days a week or maximum of one day a week, respectively. Since gender differences represent a biological variable in aging and we had limited sample size, we choose to use cells from only men for this flight and ground experiment. We collected cells from females to use in future experiments. Muscle precursor cells derived from the donor tissue samples were then purified for CD56^+^ selection, a cell surface marker expressed in myogenic cells (Fig. 1A). In this communication, we report on the differences in gene expression profiling between the flight YA- vs OS-derived myobundles and ground YA- vs OS-derived myobundles^13^. Results from our previous work incorporating the same cell stocks indicated that the precursor cells retained aspects of the donors’ phenotype to provide distinct engineered muscle myobundles^13^. The muscle myobundles were perfused in microfluidic devices custom-designed for compatibility with flight hardware (Fig. 1B). By sending our muscle tissue chips to the ISS, we tested the hypothesis that spaceflight-induced cellular and molecular changes in the muscle cells may mimic salient features of muscle dysfunction that may be differentially pronounced in the myobundles derived from young adults compared to their older counterparts.

## Results

### Set-up and launch of skeletal muscle tissue chip payload

In our first flight under the Tissue Chips in Space program, 16 muscle tissue chips were integrated into an autonomous Cubelab™, engineered by our NASA-certified implementation partner Space Tango, and launched on SpaceX CRS-21 on December 6, 2020, to the ISS for a 10-day experiment in microgravity. In this communication, we demonstrate implementation of tissue chips for space research, describe results of transcriptomic studies collected between ground and space treated tissue chips, and share the technical advancements, challenges, and opportunities in deployment of an autonomous human muscle tissue chip payload for modeling musculoskeletal disease during spaceflight. Through iterative testing in microgravity, our tissue chip platform seeks to inform us of therapeutic targets for age-related muscle wasting on Earth. This study is an important first step to generate baseline data and establish lessons learned to incorporate into our subsequent flights under the Tissue Chips in Space multi-flight funding mechanism.

Our muscle microphysiological system (MPS) includes engineered three-dimensional myobundles perfused in a custom designed polydimethylsiloxane (PDMS) device with or without embedded electrodes, as previously described (Fig. 1B)^13^. We validated a two-stage differentiation protocol that was implemented for the flight experiment. The flight timeline is outlined in Fig. 1B. Eleven days prior to launch (L-11), eight devices were seeded with pooled myoblasts from the young athletic (YA) cohort and an additional eight devices were seeded with pooled myoblasts from old sedentary (OS) cohorts. The tissue chips were switched from growth media to stage I differentiation media two days later (L-9) and subsequently switched to stage II differentiation media and integrated into a 9U CubeLab™ three days prior to launch (L-3).

The CubeLab™ was designed to maintain environmental controls beyond facility ambient conditions and capture analytics of the tissue chips. The CubeLab™ contained the following subsystems: thermal manifold, fluidic control system, movable microscope imaging device, cold flask system to maintain media 4–8 °C, conduction incubator, control electronics, and flight computer. The fluidic architecture includes a custom-designed fluidic conveyance system with built-in valving, fluid routing, a micro annular gear pump, and an in-line flow sensor for fluid flow monitoring. Each component was selected to meet fluid flow, biocompatibility, and sterility requirements, as well as CubeLab^TM^ size and power constraints. During launch, the CubeLab™ was housed in a powered accent utility locker (PAUL) and once on the ISS, crew members installed the PAUL into an EXPRESS Rack locker, which provided mechanical, electrical, and network payload interfacing. Once installed, a pre-programmed protocol was initiated as described in Materials and Methods and the module performed autonomously and reported data daily including flow rate, environmental conditions, and images downlinked to a portal via a universal asynchronous transmitter interface.

Images of a pre-defined region of interest in each myobundle were captured using the PDMS micro post as a reference point. Cell image acquisition continued every 12 h and telemetry was downlinked daily to the portal system. We did experience heat buildup from the microscope that limited some fine tuning of images. The cold flask average temperature was 9–10 °C throughout the experiment. Telemetry from the CubeLab™ recorded an average 6.5% CO_2_, 45% humidity, and manifold temperature of 37–42 °C. Although the flow rate was set to 125 μl min^-1^, the flow sensor, a diagnostic tool used to verify the movement of fluid through the sensor, showed off-nominal flow readings between 0–500 μl/min throughout the experiment, possibly due to an air bubble in the flow sensor. Pre-flight fluidic testing confirmed that salts within the cell culture media may deposit and buildup over time on the interior flow path of the flow sensor thereby affecting the flow rate readings. Small air bubbles can also become trapped in the flow sensor path contributing to unreliable flow sensor readings.

The platinum electrodes embedded at the PDMS-media channel interface were wired to the CubeLab^TM^ circuit board. Thirty minutes of electrical stimulation of 3 V, 2 Hz, 2msec pulse was applied sequentially to four YA and four OS tissue chips. During imaging, heat spikes were detected which prevented the capture of video from the chips. The experiment was planned to continue over 14 days post launch; however, we terminated the experiment after 10 days post launch to ensure robust results since there was the possibility of air blocking the fluid flow and the CubeLab experienced temperature fluctuations while operating the optics system. Space Tango’s Mission Operations Center in Lexington, KY allows for near real-time experiment intervention during off-nominal events. This capability allowed the team to initiate the RNALater protocol manually on L + 10 and each sample was flushed with at least 0.6 ml of reagent to preserve transcriptomic material. The CubeLab™ was moved to −32 °C cold stowage until payload return.

The payload returned to Earth onboard a SpaceX Dragon capsule which splashed down on January 13, 2021. The payload was returned frozen to Kennedy Space Center (KSC) Space Station Processing Facility (SSPF), where the team removed tissue chips and fluid bags from the CubeLab^TM^. Inspection of the waste bags indicated that 10 ml of media and 6 ml of RNALater flowed through the tissue chips. Although perfusion of the tissue chips over 10 days in microgravity was less than expected, the samples were sufficiently preserved with RNALater at the termination of the experiment. The RNA quantity and quality for each of the 16 tissue chips was determined by electrophoretic separation on a bioanalyzer and the results are listed in Supplementary Table 1A. The flight samples yielded an average of 1.6 ± 0.6 μg of total RNA with average RNA integrity number (RIN) of 7.8 ± 1.0, indicating high RNA integrity and sufficient material for RNA-Seq analysis (RIN values range from 10 (intact) to 1 (totally degraded)).

Post launch, we seeded cells from the same CD56+ population into an equivalent stock of PDMS chips and recapitulated the timeline shown in Fig. 1 under equivalent flow rate, temperature and % CO_2_ in a cell incubator as a ground control as described in the Methods section. The samples were terminated with RNALater and frozen to serve as ground controls to the flight samples. Chips were thawed and lysed in RLT buffer using the equivalent procedure as the flight chips. RIN values for the ground chips are listed in Supplementary Table 1B. The ground samples provided an average of 1.3 ± 0.2 μg of total RNA with an average RNA integrity number (RIN) of 9.2 ± 0.2. Chips (4xYA and 4x OS) were electrically stimulated in the same timeframe as flight using a custom microcontroller and circuit board as described^1^. Only the non-stimulated samples were compared and reported in this communication. We have since replicated a similar experiment on SpaceX CRS-25 where all eight tissue chips were stimulated for the full 8-day duration and analysis is underway directly comparing samples in the same CubeLab^TM^ sent to the ISS or incubated on ground. With our iterative testing on the ISS, we plan to compare results from both flights. Thus, communicating our preliminary results for this first-in-class muscle MPS payload is critical in standardizing and advancing cell-based autonomous systems in low Earth orbit.

### Differential expressed genes: flight vs ground and OS vs YA

We performed transcriptomic profiling to establish differential patterns of gene expression between muscle tissue chips exposed to flight for 10 days compared to tissue chips cultured on ground. Applying a RIN number cutoff of 7.0, RNA-Seq analysis was performed on three distinct chips from each group that included the young, athletic flight group (YA FI), the young, athletic ground control (YA GC), the old, sedentary flight group (OS FI), and the old, sedentary ground control (OS GC). Chips used in the analysis are labeled in Supplementary Table 1A, B. RNA-Seq results displayed as Volcano plots demonstrated that there was a total of 154 and 255 differentially expressed genes (DEGs) in flight YA- and OS-derived myobundles compared to their control groups, respectively (2-fold change, false discovery rate (FDR) < 0.05, Fig. 1C). In the data set, 110 and 44 DEGs were down- and up-regulated in YA-derived tissue chips, respectively, in spaceflight compared to ground controls (Fig. 1C); the genes are listed in Supplementary Table 2. In addition, 155 and 100 DEGs were down- and up-regulated in OS-derived tissue chips, respectively, in spaceflight compared to ground controls (Fig. 1C); these genes are listed in Supplementary Table 3. We also compared OS- vs. YA-derived myobundles from the flight group to OS vs. YA ground controls to investigate whether differences in gene profiles between YA and OS-derived tissue chips on Earth might be altered by spaceflight. We found 46 and 31 DEGs were down- and up-regulated in OS vs YA ground controls (Fig. 1C) and 22 and 44 DEGs were down- and up-regulated in the flight samples (Fig. 1C); the genes are listed in Supplementary Tables 4 and 5. There was no significant difference in the DEGs between myobundles receiving a one-time 30 min electrical stimulation prior to RNALater addition and those not receiving electrical stimulation. Therefore, as noted above, we chose to focus bioinformatic analysis on only non-electrically stimulated flight and ground samples. This study will serve as a baseline model for comparison to future flights that will include a repeat set of non-electrically stimulated tissue chips and a set of tissue chips electrically stimulated for 30 min every 12 h for 8 days.

### Post-flight transcriptomic comparisons: flight vs ground

**Fig. 2:**
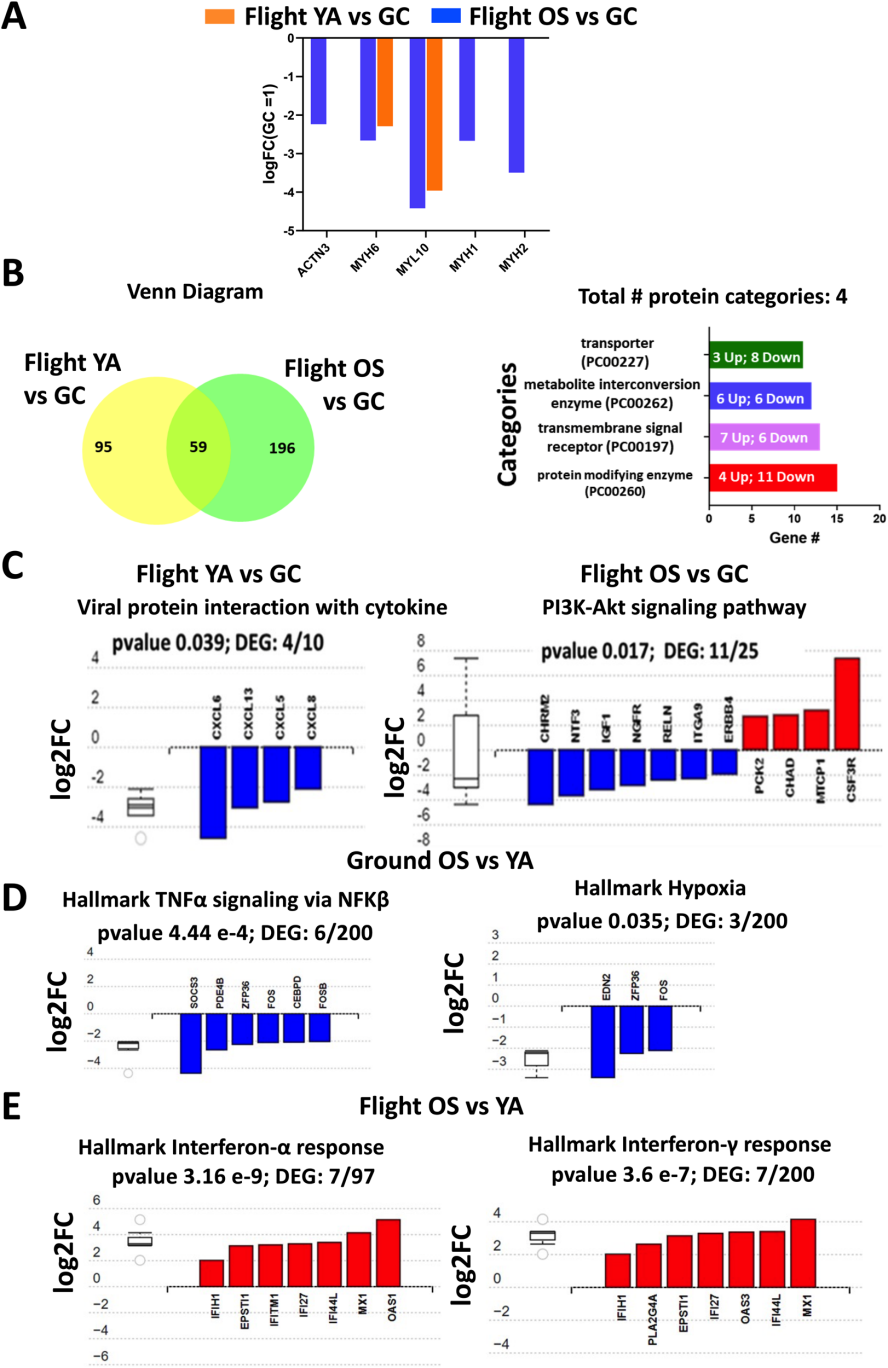
Transcriptional responses of muscle myobundles during flight and ground culturing conditions. **A** Comparative analysis of differential gene expression changes between flight and ground controls of genes involved in muscle myogenesis and contraction YA (orange bars) and OS (blue bars). **B** Panther classification system of the 59 shared genes shown in the Venn diagram for overlapping and distinct DEGs among Flight YA vs GC and Flight OS vs GC datasets. The horizontal bar chart identifies the four protein classes and relative number of DEGs of each functional class by applying a cut-off threshold of 10 genes. **C** Significantly enriched KEGG pathways in response to flight either in YA- or OS-derived myobundles relative to their respective ground controls. Significantly affected “hallmark” gene sets from the Molecular Signature Database for (**D**). Ground OS vs YA and (**E**). Flight OS vs YA (MSigDB). **C**, **D**, **E** The box and whisker plots on the left summarize the distribution of all the differentially expressed genes in the two pathways. The box represents the 25–75 quartile with a median line, the whiskers show the top and bottom 25% of the data, while the outliers are represented by doted lines. RNA-Seq analysis measured distinct three individual chips each containing one myobundle derived from 5 male donor cells pooled in equal ratios.

Previously, we reported that genes related to skeletal muscle myogenesis and myopathy are similarly upregulated in YA- and OS-derived myobundles differentiated for 2 weeks compared to their respective myoblast stages indicating transition to mature myotubes^13^. As such, we implemented the same differentiation protocol for the current experiments. Our results suggest tissue chips exposed to spaceflight may induce specific shifts in the expression of myosin heavy- and/or light-chain genes (Fig. 2A). We observed that myogenic genes α-actinin-3 (ACTN3), myosin heavy chain 1, 2, and 6 (MYH1, MYH2, MYH6), and myosin light-chain isoform 10 (MYL10) were significantly downregulated in flight OS-derived myobundles compared to ground controls (Fig. 2A). Of these genes, MYH6 and MYL10 were also downregulated in flight YA-derived myobundles versus ground controls. Studies conducted on astronauts have shown that skeletal muscles remodel their metabolic profile and transition fiber types to allow for metabolic adaptation to the spaceflight environment^20–22^. In humans, type 1 fibers are oxidative, and type 2 A and 2X fibers primarily rely upon glycolytic metabolism^23^. Microgravity stimulates slow-to-fast fiber transition^24,25^, whereas age and exercise induce fast-to-slow fiber transition^26,27^. In our in vitro model, these alterations in myosin gene expression composition are pronounced in the OS tissue chips, indicating a down-regulation of mRNA levels of MYH1 (FDR = 4.3 e^−13^) and MYH2 (FDR = 6.7 e^−24^) by 2.6 and 3.5-fold. These genes encode for Myosin heavy chain-1 (fast type IIX muscle fibers) and Myosin heavy chain-2 (fast type IIA muscle fibers), respectively. In addition, the fast type II fiber-related gene, ACTN3 (FDR = 1.9 e^−5^) is downregulated by 2.2-fold (Fig. 2A). Both YA- and OS-derived myobundles showed down-regulation of the MYH6 gene, present in slow-twitch type I fibers, by 2.3- (FDR = 8.6 e^−7^) and 2.7- (FDR = 3.3 e^−9^) fold, respectively. The downregulation of MYL10, which enables calcium binding in the motor protein, in both YA- and OS-derived myobundles also supports a shift in fiber type. Overall, these results suggest that the spaceflight may affect overall fiber type transition predominately in the myobundles derived from the aged cohort. Myobundles derived from the young cohort may undergo transition from slow to fast. This may be confirmed in our follow-on flight opportunity.

Applying the Panther classification system^28^, we observed 59 shared genes between the flight YA- and OS-derived tissue chips compared to ground controls (Fig. 2B, Venn diagram). The analysis identified four strongly impacted protein categories (log2 FC ≥ ± 2) including protein modifying enzyme class, metabolite interconversion enzyme class, transmembrane signal receptor class, and transporter protein class with 15, 13, 12 and 11 significantly modulated genes, respectively (Fig. 2B, total protein categories). The values for these gene targets are displayed as heatmaps in Supplementary Fig. 1A–D.

Skeletal muscle is characterized by elevated utilization of glucose and high fatty acid oxidation rates^29^. Our data in Supplementary Fig. 1A–D suggest that genes encoding for enzymes involved in the regulation of glucose are dysregulated in flight samples, regardless of the young or old phenotype. These results suggest metabolism remodeling. In the protein modifying enzyme class, we observed the downregulation of pyruvate dehydrogenase kinase 4 (PDK4), a key metabolic enzyme that regulates muscular pyruvate metabolism by inhibiting pyruvate dehydrogenase (PDH) by 3.3- and 2.8-fold in YA- and OS-derived tissue chips, respectively, when exposed to flight compared to ground controls (Supplementary Fig. 1A). PDK4 mRNA are markedly increased in human skeletal muscle during prolonged exercise^30^. The flight samples exhibit decreased PDK4 mRNA in the microtissues, subsequently inducing entry of carbohydrate-derived pyruvate into the Krebs cycle and undergoing oxidative phosphorylation with ATP production.

Furthermore, YA and OS flight microtissues were characterized by increased mRNA levels of genes encoding for key rate-limiting enzymes involved in glycolysis as shown in the metabolite interconversion enzyme protein class (Supplementary Fig. 1B). Phosphofructokinase (PFKP), a key enzyme in glycolysis, was up-regulated by 2.5- and 2.0-fold in YA- and OS-derived tissue chips, respectively, in flight compared to ground controls. Phosphoenolpyruvate carboxykinase 2 (PCK-2) gene, which encodes for an enzyme involved in glucose biosynthesis, was also up-regulated by 2.0 and 2.7-fold in YA and OS flight samples, respectively, compared to ground controls. These results indicate the potential response of the flight samples to increased glucose metabolism.

Other enzyme-encoding genes in the metabolite interconversion enzyme protein class similarly up-regulated in both tissue chip sets in flight compared to ground controls included nitric oxide synthase (NOS1) and arginase 2 (ARG2) genes, two enzymes that metabolize L-arginine (Supplementary Fig. 1B). Evidence indicates that L-arginine protects muscle cells from wasting in vitro in an mTORC1-dependent manner^31,32^ by promoting protein anabolism in myocytes through the involvement of the nitric oxide (NO) and mTOR/p70S6K signaling pathways^33,34^. Also, NO has been associated with skeletal muscle-wasting diseases, sarcopenia, and cachexia^35^ and activation of NO during muscle injury is critical in the early phases of the skeletal muscle repair processes^33,36^. However, the role of NO in myocyte protein synthesis under microgravity conditions needs to be elucidated.

One of the top 5 significantly upregulated genes in both YA (5-fold) and OS (7.3-fold) flight samples compared to ground controls encodes granulocyte colony-stimulating factor receptor 3 (CSF3R) in the transmembrane receptor class (Supplementary Fig. 1C, Supplementary Tables 2 and 3). CSFR is expressed in skeletal muscle during development and in adults^37,38^. The receptor plays pivotal roles in skeletal myocyte development, regulation of muscle regeneration and is increased in injured skeletal muscle. Treatment with CSF has been shown to enhance muscle regeneration processes by promoting the proliferation of satellite cells^38^. It is not clear if high CSF3R levels may dysregulate muscle regeneration pathways in humans. Our data suggests a potential modulation of the CSF/CSFR axis in the regulation of muscle volume in the tissue chips exposed to flight.

On the other end of the spectrum, members of the transporter class including those from the potassium and sodium voltage gated channel families are significantly downregulated in flight samples compared to ground controls (Supplementary Fig. 1D). Members of these ion channel genes are expressed in the plasmalemma, T tubules, and sarcoplasmic reticulum of skeletal muscle responsible for controlling muscle fiber electrical properties and signal transduction of the action potential^39^. The potassium channel subfamily A member 10 (KCNA10) channel is downregulated in both YA (2.3-fold) and OS (4.6-fold) flight samples compared to ground controls and the sodium channel alpha subunit 3 (SCN3A) channel is downregulated in YA (2.6-fold) and OS (3.8-fold) flight samples compared to ground controls (Supplementary Tables 2, 3). The alpha subunits 2 and 7 are also downregulated in OS-derived flight samples by 2.4- and 2.5-fold, respectively, indicating the downregulation of muscle fiber activity may be more pronounced in the aged-derived myobundles. The tissue chips we are investigating are not undergoing electrical stimulation. In subsequent flights, we have applied electrical stimulation to half of the flight samples and the resulting data may be compared to results from our first flight to understand how microgravity affects the electrical properties of the myobundles.

Functional enrichment using Kyoto Encyclopedia of Genes and Genomes (KEGG) pathway^40^ analysis in YA-derived myobundles revealed enrichment of downregulated CXC chemokine signaling genes (DEGs = 4/10; *P* = 0.039, Fig. 2C, left). In healthy skeletal muscle, the chemokine expression profile is low, however, many chemokines are induced or upregulated in dystrophic muscle^41–43^. In addition, we observed only in YA-derived flight samples compared to ground controls, downregulation of JAK-STAT signaling including decreased expression levels of the following: (1) transcription factor Jun B proto-oncogen (JUNB), (2) the suppressor of cytokine signaling 1 (SOCS1) and (3) the cytokine inducible SH2 containing protein (CISH) by 2.13-, 2.8- and 4.6-fold, respectively (Supplementary Table 2). Since our differentiated muscle myobundles do not contain a satellite cell niche, the downregulation of the chemokine signaling, and JAK/STAT pathway indicates a compensatory mechanism to protect against muscle wasting. JunB has been suggested to play a role in both the maintenance and hypertrophy of skeletal muscle mass and to stimulate protein synthesis independently of Akt and mTOR^44^. Several studies have suggested that the activity of JunB is finely regulated in skeletal muscle. For instance, the expression of JunB (AP-transcription factor subunit), can be induced by exercise^45^. Our findings are consistent with the observations that JunB expression is decreased during various types of atrophy^46,47^.

In the flight OS-derived myobundles, we did not observe modulation of the CXC chemokine pathways. Rather, the KEGG pathway analysis revealed that DEGs were enriched in the phosphoinositide 3-kinase (PI3K)/threonine protein kinase B (Akt) signaling pathway (DEGs = 11/25; *P* = 0.017, Fig. 2C, right). The PI3K/Akt pathway is a predominant pathway controlling skeletal muscle metabolism. Exogenous insulin-like growth factor-1 (IGF-1) targeting PI3K/Akt is known to increase skeletal muscle protein synthesis via activation of Akt phosphorylation and mTOR activation and has emerged as a potential target for mitigating skeletal muscle loss due to microgravity^48^. IGF-1 expression was downregulated 3.2-fold in OS flight samples compared to ground controls (Fig. 2C, right, Supplementary Table 3). The dysregulation of IGF-1 downstream signaling pathways and the reduction of myosin content in OS flight samples suggest a negative impact on muscle microtissue health specific to muscle cells from older adult-derived myobundles.

Next, we evaluated gene enrichment and functional annotation of DEGs using the Database for Annotation, Visualization, and Integrated Discovery (DAVID)^49^ bioinformatic tool. The relevant Gene Ontology (GO) annotations were determined by filtering each contrast with *p*-values ≤ 0.05, logFC ≥ ±2 and DEG per group ≥14. Functional annotation of proteins encoded by DEGs with increased or decreased enrichment compared with their controls were classified according to their associated biological processes, molecular functions, and cellular components. Relevant GO ontological groups were visualized using bubble plots presented in Supplementary Fig. 2A, B and a summary of the detailed results is listed in Supplementary Tables 4 and 5 for YA flight versus ground and OS flight versus ground, respectively. Analysis of cellular component in YA and OS revealed the most significant terms are “GO:0016021~integral component” of membrane and “GO:0005886~ plasma membrane”, respectively. Regarding the integral component of membrane in YA, a total of 18 DEGs were up- and 33 were down-regulated (Supplementary Table 4). In the OS, a total of 38 were up- and 55 were down-regulated, respectively within the plasma membrane term (Supplementary Table 5). At the level of molecular function, the GO analysis showed the most significant terms in YA and OS are “GO:0042802~ identical protein binding” and “GO:0005509~ calcium ion binding” with 5 up- and 13 down- and 7 up- and 18 down-regulated genes, respectively. Finally, biological process analysis, DEGs are mainly enriched in “GO:0007165~ signal transduction” with 10 up- and 13 down-regulated genes.

### Post-flight transcriptomic comparisons: OS vs YA

Finally, we compared differences between OS- vs. YA-derived myobundles from the flight group to OS vs. YA ground controls to investigate decreases in gene profiles between OS and YA-derived myobundles due to spaceflight. Gene set lists were derived using Hallmark gene sets deposited in the molecular signatures database (MsigDB). When OS myotubes were compared with YA in ground conditions, we found 6 and 3 significantly down-regulated DEGs that are assigned to HALLMARK gene sets that regulated TNFα signaling via NFK (*p*-value ≤ 4.44 e-4) and Hypoxia (*p*-value ≤ 0.035) signaling, respectively (Fig. 2D). Interestingly, when we compared OS vs YA cultured in spaceflight conditions, the analysis showed two HALLMARK gene sets expressed in response to interferon-α (*p*-value ≤ 3.16 e-9) and -γ (*p*-value ≤ 3.6 e-7) with 7 significantly up-regulated DEGs (Fig. 2E).

Venn-diagram analysis (Supplementary Fig. 3A, B) showed 14 and 4 shared DEGs were up- and down-regulated, respectively, when OS vs YA ground samples were compared with OS vs YA flight samples. 17 and 42 genes were up- and down-regulated only in ground OS vs YA samples and 32 and 19 genes were up- and down-regulated only in the flight OS vs YA samples (Supplementary Fig. 3A, B and Supplementary Tables 6 and 7). These genes were further classified by their function using Panther classification system (https://www.pantherdb.org/). The analysis revealed that the 18 shared DEGs were identified in two strongly impacted protein categories (log2 FC ≥ ± 2) including proteins involved in “metabolite interconversion enzyme” and “protein modifying enzyme” and other unclassified with 3, 4, and 4 significantly modulated genes, respectively (Supplementary Fig. 3C). The 59 and 48 DEGs from ground and flight conditions by Panther classification system were classified into 7 and 6 protein categories, respectively, which are displayed in Supplementary Fig. 3D and E. The DEGs gene targets were predominantly classified as “metabolite interconversion enzyme”, “transmembrane signal receptor”, “transporter”, “gene-specific transcriptional regulator” in both conditions.

## Discussion

Human pathophysiological adaptations to the microgravity environment include loss of skeletal muscle mass, decreased muscle force, fiber-type shift, and metabolic alterations that put astronauts at risk for injury during spaceflight operation and are of great concern for long-term space missions^50^. The molecular mechanisms triggering skeletal muscle remodeling and dysfunction remain to be fully elucidated, however, the findings from studies in humans, rodents, and in vitro systems in simulated and real microgravity have highlighted key factors that contribute to spaceflight induced muscle atrophy. These mechanisms include protein catabolic effects and are critical to microgravity-induced muscle tissue loss^51,52^. Several questions remain with respect to how these underlying mechanisms contributing to muscle loss in space mimic the salient features of age-related muscle dysfunction. For example, it is important to understand the molecular events controlling metabolic adaptations of muscle cells to microgravity and how the expression levels of biomarkers and mechanisms are modulated in microgravity to regulate skeletal muscle differentiation and biomechanics. As more cell and tissue-based experimentation is performed in the microgravity environment, we can appreciate the impact of microgravity at the tissue level to reveal underlying mechanisms contributing to accelerated decline in muscle properties of clinical relevance. These in vitro disease models may be used as space-based platforms to develop countermeasures and biomarkers to preserve skeletal muscle health particularly in the aging population, diagnose the disease early, and ameliorate the healthcare burden associated with sarcopenia. Thus, an aging disease model that recapitulates age-related muscle dysfunction accelerated by microgravity is essential for functional analysis and has the advantage of excluding the effects of systemic factors and allowing for evaluation of molecular mechanisms at the cellular level. Future flights may offer larger sample sizes to evaluate both male and female cells and individual donors. This study was limited in sample size, and we choose to use pooled male cells and cannot address the potential effects of sex as a biological variable.

The results generated from our first flight, under the Tissue Chips in Space program, have demonstrated tissue-on-chip experimentation on the ISS and advanced on-orbit tissue chip imaging. Overall, we demonstrate that our engineered 3D skeletal muscle MPS holds promise to model skeletal muscle dysfunction and reveal corresponding molecular pathways modulated during spaceflight that may mimic manifestation of age-related muscle atrophy. Collectively, our data identified genes belonging to protein modifying enzyme, transmembrane signal receptor, metabolite interconversion enzyme, and transporter gene classes that are modulated in tissue chips differentiated in flight compared to ground controls. The downregulation of key genes that encode for skeletal specific structural proteins, MYH1, MYH2, MYH6, MYL10, ACTN3, particularly in the OS-derived flight samples compared to ground controls, suggests impaired skeletal muscle differentiation and sensitization of the OS-derived flight samples and suggests fiber type switch in both OS- and YA-derived flight samples. In addition, in the OS flight samples, the PI3K/Akt pathway is modulated, suggesting activation of pathways controlling muscle metabolism and protein synthesis. Finally, the significant upregulation of CSF3R in myobundles from both cohorts during spaceflight suggests that inflammatory pathways are induced.

When we directly compare ground OS vs YA to flight OS vs YA, we detect fewer DEGs in the flight samples compared to ground samples (66 DEGs vs 77 DEGs, Fig. 1C). Interestingly, TNFα signaling is downregulated in ground OS cohort and interferon α and γ signaling is upregulated in flight OS cohort in relation to the YA cohort. This further suggests that the OS-derived myobundles are more sensitive to dysregulation of immune mediators in spaceflight. Lastly, in YA flight samples, cytokine signaling is suppressed as seen by the KEGG pathway analysis and the downregulation of CXC chemokine receptors supporting a skewed cytokine profile in spaceflight^5^. These results support that gene differences observed between YA- and OS-derived myobundles on Earth are altered by spaceflight.

Our team learned valuable information regarding subsystem performance that we have implemented on Space-X CRS 25 (data analysis in progress) to advance the lab-on-chip model and functionality and provide comparative omics analysis. Data collected from SpaceX CRS-25 will include repeat experimentation of tissue chips derived from the same lot of YA- and OS-pooled cells and non-electrically stimulated over the same time course shown in Fig. 1B. An additional set of differentiated tissue chips are electrically stimulated every 12 h for 8 days once installed on the ISS.

On Earth, tissue chip platforms are innovative technologies to advance insights into human biology early in the drug discovery process. Translation of tissue chip platforms to in-space versions and validating the technological approach is exciting, immensely challenging and a longer-term commitment. In our first experiment, we were successful in operating the CubeLab^TM^ autonomously over several days and capturing images of the tissue chips. We maintained CO_2_ levels and sterility in the CubeLab^TM^ environment. We encountered challenges in controlling heating, cooling, and humidity in the closed environment to mimic a cell incubator. High humidity levels contribute to corrosion of electronics, but too low humidity levels contributed to evaporation in the microfluidic devices and introduction of air bubbles which interfered with the flow sensor readings. We also determined that the microscope computer processor needed to be mounted more securely to the top of the CubeLab^TM^ to ensure proper heat transfer to the external structure of the CubeLab^TM^. The team does not believe spaceflight contributed to any hardware issues because the PAUL provides vibration dampening during launch. The team decided to terminate the experiment earlier than planned and successfully initiated the RNALater step remotely, which we believe preserved the biology and allowed us to collect and analyze the data communicated here. Despite the hurdles in integrating muscle microtissues into a miniaturized, autonomous laboratory, the ability to modify the experimental protocol remotely to maximize scientific performance and preserve biology is a major advantage. The other major advantage is that the experiment did not take crew time to complete.

The CubeLab^TM^ system is complex and each subsystem (i.e., thermal management, fluid delivery system, microscope) is designed to perform its specific function independently and ensure no interferences transfer within the closed system to negatively affect the performance of other subsystems. We performed several experimental verification tests (EVTs) to validate subsystems operation and informed multiple design changes from the physical shape of the stimulation board to the mounting and perfusion of the tissue chips. One of the challenges that we encountered was that fully integrated CubeLab^TM^ testing was minimal due to timing constraints including flight manifest changes, scheduling challenges, and funding timelines, as well as facilities access and significant supply chain issues leading up to launch during the COVID-19 pandemic (March 2020 - December 2020).

An aspect of our CubeLab^TM^ experiment is that it housed microtissues derived from phenotyped donors and we can compare effects on gene expression in myobundles exposed to spaceflight with genetically matched ground control samples, providing an opportunity for future precision medicine experiments to be conducted in the space environment. Our experimentation in space offers integration of tissue chips into an autonomous laboratory. Eventually, biomedical science payloads need to work onboard manned and unmanned space vehicles and be conducted autonomously for in-space operation with minimum-as-possible interaction by the crew away from the ground control center. The autonomous end-to-end experimentation represents a significant advantage for future flights. The CubeLab^TM^ provides the ability to control experimental conditions and capture real-time analytics that advance our capabilities to perform non-invasive, long duration experiments and collect normative data sets between flight and ground. A current limitation of the CubeLab^TM^ is that the unit is nonmodular and termination of samples at early timepoints such as immediately following launch to investigate the effects of launch stress on the tissue chips is not possible because this requires different temperature storage conditions.

In summary, our scientific study helps push the boundaries of tissue engineering conducted on the ISS to study age-related alterations at the cellular and molecular level in microgravity. Thus, communicating results of these first experiments is critical for standardization and adaptation of these technologies for space use. In addition, iterative testing in space and acknowledging lessons learned will increase robustness, affordability, and accessibility of conducting tissue chip platforms in space. Preliminary flight experiments point toward the utility of space based MPS platforms in contributing towards more accurately modeling age-related and chronic diseases. Tissue engineering studies conducted in microgravity may providing insights to disease progression otherwise not obtainable on Earth. Continued access to space and iterative testing of tissue-on-chip payloads to be performed on the ISS or on long duration lunar missions may potentially lead to therapeutics to counteract these health conditions on Earth.

## Methods

### Myoblast isolation

Vastus lateralis biopsies were obtained from volunteers, phenotyped by age and physical activity at the Translational Research Institute at AdventHealth, Orlando. All participants provided written informed consent, and the study protocol was reviewed and approved by the institutional review board at AdventHealth, Orlando (IRBNet #554559). Cells were received at the University of Florida through a signed Material Transfer Agreement. The active group engaged in structured exercise at least 3 times per week and the sedentary group exercised one or less times per week. Satellite cells from individual donors were isolated, pre-plated to remove fibroblasts and pooled in equal ratios to generate a mean cell stock of young, active cohort (YA; 21–40 years, male, *n* = 5) and old, sedentary cohort (OS, 65–80 years, male, *n* = 5) prior to cryopreservation and shipment to the University of Florida through a Material Transfer Agreement and in the absence of any patient identifiable information. Muscle myoblasts were thawed and then cultured on T75 flasks coated with collagen I in Skeletal Muscle Growth medium (PromoCell, Heidelberg, Germany) to confluence. Immunopurification of mononuclear myoblasts was performed using mouse monoclonal 5.1H11 anti-CD56 antibody (DSHB Hybridoma Bank, Iowa City, IA). CD56+ myoblasts were confirmed by FACS as described^13^.

### Preparation of bioengineered myobundles (flight and ground)

Custom microfluidic chips made from polydimethylsiloxane (PDMS) and containing two platinum 22-gauge electrode leads were obtained from Micro-gRx, INC, (Orlando, FL), sterilized in ethanol and autoclaved at 150 °C for 30 min prior to use. Cell seeding was performed at NASA’s Kennedy Space Station Processing Facility (SSPF) for flight and at the University of Florida laboratory for the ground test. Injectable hydrogel mixtures (3.3 mg/mL rat tail collagen I, and 22% (v/v) Matrigel) were combined with the 5-donor pooled YA- or the 5-donor pooled OS-derived CD56+ enriched myoblasts. The cell-laden hydrogels were injected into the PDMS chips to a final cell density of 15 and 20 million cells/mL for pooled YA- and pooled OS-derived cells, respectively. Cells were allowed to polymerize at 37 °C for 60 min and seeding ports were sealed using polylactic acid (PLA, Makerbot, Brooklyn, NY) plugs. Chips were perfused with degassed Skeletal Muscle Growth media supplemented with 0.1 mg/ml Primocin (InvivoGen, San Diego, CA) at a constant flow rate of 1 mL/h at 37 °C and 5% CO_2_ using programmable syringe pumps (Cole Palmer, Vernon Hills, IL). After 48 h, the culturing medium was changed to degassed differentiation media I (MEM-α, 0.5% (v/v) ITS, 2%(v/v) B27,10 μM DAPT, 1 μM Dabrafenib, 20 mM HEPES, pH 7.3 and, 0.1 mg/mL Primocin) and delivered at an intermittent rate of 125 μl min^−1^ for 1 min every 8 h for 6 days before integrating the chips for flight or ground studies.

### Tissue chip flight payload assembly

The sterile CubeLab™ fluidic system was flushed with sterile Phosphate-Buffered Saline (PBS) followed by priming with degassed differentiation media II (MEM-α, 0.5% (v/v) ITS, 2%(v/v) B27, 20 mM HEPES, pH 7.3 and, 0.1 mg ml^-1^ Primocin). PBS effluent was collected, plated, and incubated at 37 °C for 5 days followed by RT (20–25 °C) for 5 days (sterile PBS was plated as control). No colonies were present confirming sterility of the fluidic system. 48 h prior to launch (L-2), tissue chips were exchanged with 1 ml media, disconnected from the syringe pump, inspected under a 10x microscope and 16 chips were integrated into a custom designed 9U CubeLab™ (Space Tango, Lexington KY). Of the 16 chips, there were eight chip replicates of the YA mean cohort and eight chip replicates of the OS mean cohort (8xYA and 8x OS). On L-3, flight chips were secured PDMS-side down onto fluid connection ports which allowed for fluid-tight mating to the manifold chip posts via press fit connections. A low pulsatile annular gear pump and Takasago-modified valves, controlled fluid to each chip. Two thermoelectric coolers located on the opposite side of the chips ensured consistent temperature (37–39 °C). The stimulation circuit was connected to eight chip’s stimulation leads via blue wire and connectors custom to the stimulation board mounted on one side of the manifold. 200 ml of chilled, degassed media II was added to the source bag and placed into the cold flask (4–8 °C). Chilled RNALater (20 ml) was added to a second source bag and placed into the cold flask (4–8 °C). Fluid from each chip (200 ml) was routed to general waste. The payload was sealed, purged with 5% CO_2_ using an air gas canister and the thermal protocol was initiated to bring the manifold to 37 °C. Prior to handover, a leak test was performed to verify pressure integrity and the CubeLab™ was integrated into the powered accent utility locker (PAUL). Once in the PAUL, the camera system was initiated and calibrated for each chip position and the *Media Exchange-Waste* sequence I (outlined below) was initiated prior to handover to NASA at L-1.

### On-orbit payload operation

After docking (L + 2), the CubeLab™ was installed by crew members and operated from within the PAUL. After install, the following protocols were initiated: (1) The *Media Exchange-Waste sequence 2*. During this event, media was flowed to each chip every 8 h at 125 μl min^-1^ for 1 min and output volume was sent to general waste. (2) The *Z-Stack* event. During this event, the microscope (10x magnification) was moved to chip *N* and captured six phase contrast slices at 12 μm increments at each chip’s pre-defined position immediately before and after each Media-Exchange-Waste event. (3) The *Stimulation & Video* event was initiated on L + 9. During this event, 8 chips (4xYA and 4xOS) were electrically stimulated. The remaining 8 chips (4xYA and 4x OS) were non-stimulated. The intended program involved moving the microscope to chip *N*’s ideal position and recording video at 25 fps for 40 s (pre-stimulation phase), then at the 41 s mark after video initiation, a stimulation waveform (3 V, 2 Hz, 2msec) on chip *N* was elicited and video was to be recorded for an additional 40 s (stimulation phase). Finally, at the 82 s mark after video initiation, the stimulation waveform ceased, and video was to be recorded for 40 s (recovery phase) for total of 120 s of video for each of the 8 chips. The program was to be repeated every 24 h for 5 days. The executed program included stimulation of the 8 chips for 30 min on one day prior to initiation of fixation. (4) The *Fixation* event. During this event, RNALater was initiated to each chip at 125 μl min^-1^ for 5 min at termination of the experiment. Crew members removed the CubeLab™ from the PAUL and transferred to the ICEBERG cold stowage asset, at −35 °C. Continuous telemetry from the CubeLab™ environment was recorded in the customer dashboard portal. Z-stacked images of each chip per day and the stimulation video were downlinked to the portal system. An open-source digital image correlation (DIC) algorithm based in MATLAB (Mathworks, Natick, MA) was used to quantify displacements at the region of interest in the tissue chip as previously described^1^.

### Post-flight RNA extraction

Payload splashdown from SpaceX CRS-21 occurred on January 14, 2021, and the CubeLab™ was transferred from a Double Cold Bag (conditioned at −32 °C) to a −80 °C freezer at NASA’s Kennedy SSPF processing laboratory where the CubeLab™ was de-integrated and chips were removed and immediately processed for RNA isolation. Flight chips in RNALater were lysed in RLT buffer (Qiagen, Germantown, MD) prepared with B-mercaptoethanol. RNA isolation was then performed using RNeasy Plus Mini Kit according to manufacturer’s instructions. The integrity of the extracted RNA was assessed by Agilent 2200 Tapestation system (Agilent, Santa Clara, CA). Only the non-stimulated samples were compared and reported in this communication. RIN values for the flight chips are listed in Supplementary Table 1A.

### Asynchronous ground experiment

The ground experiment was performed at the University of Florida approximately 30 days following the return and de-integration of the flight study. Aliquots from the same lot of pooled YA and pooled OS cells (8x YA and 8x OS) used for flight were used for the ground study and the 16 tissue chips were seeded and differentiated as described in the Preparation of Bioengineered Myobundles (Flight and Ground) section. On the equivalent of day “L-3”, ground chips remained connected to the syringe pumps in the cell incubator (37 °C and 5% CO_2_) and differentiation media I in 3 ml syringes were changed to degassed differentiation media II and delivered to the ground chips every 8 h at 125 μl min^-1^ for 1 min to mimic the integration of the flight chips into the CubeLab^TM^ and the switch from media I to II. On the equivalent day as “L + 9”, 8 chips (4xYA and 4xOS) were electrically stimulated with wave form 3 V, 2 Hz, 2msec for 30 min using a custom microcontroller and circuit board as described^1^. No video was collected as we did not have the capability to record inside the incubator. The remaining 8 chips (4xYA and 4xOS) were non-stimulated. On the equivalent day as “L + 10”, the tissue chips were disconnected from the syringe pump and 1 ml of RNALater was manually added to the chips to mimic flight samples and stored at −20 °C for more than 24 h.

### RNA sequencing analysis

Libraries of extracted RNA samples were built using the NEBNext® Ultra™ Directional RNA Library Prep Kit for Illumina (NEB, USA) RNA sample preparation kit and yielded fragments with 220–700 base pairs. The qualified fragments were ligated with 60 adapters, amplified, and submitted for sequencing by Illumina NovaSeq 6000 (Illumina, San Diego, CA) to generate paired end reads with a length of 150 bases. The input sequences were trimmed using trimmomatic. Quality control was performed before and after trimming using FastQC (v 0.11.4) and MultiQC^53^ and a total of 50 million reads were generated for each sample yielding coverage in the range of 118 to 220 bases for each sample, Then the input sequences were aligned to the transcriptome using the STAR aligner, version 2.7.9a^54^. Transcript abundance was quantified using RSEM (RSEM v1.3.1)^55^, and genes with insufficient average counts were excluded from further statistical analysis. Differential expression analysis was performed using the DESeq2 package^56^, with an FDR-corrected P-value threshold of 0.05. The results were further filtered to extract transcripts showing a 2.0-fold change (log2FC) in either direction.

### Differential expression and functional annotation analysis of RNA-seq data

Differentially expressed genes (DEGs) from YA- and OS flight samples were normalized to the matching ground control group and reported as log2 of the fold change (log2FC). Fold induction values were averaged for all experiments performed as experimental triplicates for each cohort. RNA-seq data were analyzed by iPathwayGuide (Advaita Bioinformatics: http://www.advaitabio.com/ipathwayguide.html) by identifying significantly impacted signaling pathways. Volcano plots, which rely on double filtering criterion and display unstandardized signal log2FC against noise-adjusted/standardized signal FDR-corrected *P*-value, were used to display up- and down-regulated DEGs. Gene Ontology (GO) and Pathway enrichment analysis were performed by comparing DEGs with Kyoto Encyclopedia of Genes and Genomes (KEGG) databases where *p* < 0.05 was statistically significant. Database for Annotation, Visualization, and Integrated Discovery database (DAVID, https://david.ncifcrf.gov/) was used to determine the biological meaning to a given set of DEGs and categorized them by GO-molecular function, GO-biological process, and GO-cellular component. In DAVID database, Fisher’s Exact test is adopted to measure the gene-enrichment in annotation terms. Fisher’s Exact *p*-values are computed by summing probabilities p over defined sets of tables (*Prob* = *∑Ap*) and looks for any functional enrichment against a background that consist of all *Homo Sapiens* genes. Each GO term is used to describe the features of genes and gene products. For each Gene Ontology (GO) term, the number of DEGs annotated to the term is compared against the number of genes expected by chance. Significance of each GO term was assessed using the default homo sapiens GO annotation as background. A GO term was considered statistically significant at FDR-corrected *P* ≤ 0.05. IPathwayGuide uses an overrepresentation test, based on a hypergeometric distribution, to compute the statistical significance of observing more than the expected number of DEGs. Significance of GO terms were assessed using the default *Homo Sapiens* GO annotation as background. A GO term was considered statistically significant at FDR-corrected *P* ≤ 0.05. Next, Protein Analysis Through Evolutionary Relationships (PANTHER v.13.0, www.pantherdb.org) Classification System was used to perform protein-encoding genes functional classification and provide information about protein families. PANTHER uses an overrepresentation test, FDR-corrected *P* ≤ 0.05. The analysis was applied only to DEGs with cut-off of adjusted *p*-value of 0.05 and fold-change ≥ ±2.

### Statistical analysis

Quantification methods are described in Methods Section. Specific tests used are indicated in the Figure legends. P values for significant differences are indicated in the graphs. All graphs show the individual data points used in the analysis.

### Reporting summary

Further information on research design is available in the Nature Research Reporting Summary linked to this article.

### Supplementary information

Supplementary Material

Reporting Summary

## Data Availability

All data generated during this study are either included in the manuscript and its Supplementary files or are available at the Gene Expression Omnibus (GEO) database (accession number GSE234465). Precursor cells from patient biopsy samples were obtained from AdventHealth Orlando through a Material Transfer Agreement to the University of Florida with restrictions for sharing with a third party.

## References

[1] Sharma A (2022). Biomanufacturing in low Earth orbit for regenerative medicine. Stem Cell Rep..

[2] Capri M (2023). Long-term human spaceflight and inflammaging: does it promote aging?. Ageing Res. Rev..

[3] Sy MR, Keefe JA, Sutton JP, Wehrens XHT (2023). Cardiac function, structural, and electrical remodeling by microgravity exposure. Am. J. Physiol. Heart Circ. Physiol..

[4] Low LA, Giulianotti MA (2019). Tissue chips in space: modeling human diseases in microgravity. Pharm. Res..

[5] Crucian BE (2018). Immune system dysregulation during spaceflight: potential countermeasures for deep space exploration missions. Front. Immunol..

[6] Fabre KM (2019). Utilizing microphysiological systems and induced pluripotent stem cells for disease modeling: a case study for blood brain barrier research in a pharmaceutical setting. Adv. Drug Deliv. Rev..

[7] Ewart L (2018). Application of microphysiological systems to enhance safety assessment in drug discovery. Annu Rev. Pharm. Toxicol..

[8] Thomas D, Choi S, Alamana C, Parker KK, Wu JC (2022). Cellular and engineered organoids for cardiovascular models. Circ. Res..

[9] Baregamian N (2023). Engineering functional 3-dimensional patient-derived endocrine organoids for broad multiplatform applications. Surgery.

[10] Bae M, Yi HG, Jang J, Cho DW (2020). Microphysiological systems for neurodegenerative diseases in central nervous system. Micromachines (Basel).

[11] Rubiano A (2021). Characterizing the reproducibility in using a liver microphysiological system for assaying drug toxicity, metabolism, and accumulation. Clin. Transl. Sci..

[12] Han JJ (2023). FDA modernization Act 2.0 allows for alternatives to animal testing. Artif. Organs.

[13] Giza S (2022). Microphysiological system for studying contractile differences in young, active, and old, sedentary adult derived skeletal muscle cells. Aging Cell.

[14] Fielding RA (2011). Sarcopenia: an undiagnosed condition in older adults. Current consensus definition: prevalence, etiology, and consequences. International working group on sarcopenia. J. Am. Med Dir. Assoc..

[15] Coen PM, Musci RV, Hinkley JM, Miller BF (2018). Mitochondria as a target for mitigating sarcopenia. Front. Physiol..

[16] Morley JE (2016). Pharmacologic options for the treatment of sarcopenia. Calcif. Tissue Int..

[17] Holloszy, J. O. The biology of aging. *Mayo Clin. Proc.* **75 Suppl:S3-8,** discussion S8-9 (2000). 10959208

[18] Melton LJ (2000). Epidemiology of sarcopenia. J. Am. Geriatr. Soc..

[19] Scott JM (2023). Effects of exercise countermeasures on multisystem function in long duration spaceflight astronauts. NPJ Microgravity.

[20] Cannavo A (2022). Are skeletal muscle changes during prolonged space flights similar to those experienced by frail and sarcopenic older adults?. Life (Basel).

[21] Murgia M (2022). Signatures of muscle disuse in spaceflight and bed rest revealed by single muscle fiber proteomics. PNAS Nexus.

[22] Fitts RH, Riley DR, Widrick JJ (2000). Physiology of a microgravity environment invited review: microgravity and skeletal muscle. J. Appl. Physiol. (1985).

[23] Murgia M (2021). Protein profile of fiber types in human skeletal muscle: a single-fiber proteomics study. Skelet. Muscle.

[24] Bagley J, Murach K, Trappe S (2012). Microgravity-induced fiber type shift in human skeletal muscle. Gravitational Space Biol..

[25] Shenkman BS (2016). From slow to fast: hypogravity-induced remodeling of muscle fiber myosin phenotype. Acta Nat..

[26] Hayashi C, Ogata S, Okano T, Toyoda H, Mashino S (2021). Long-term participation in community group exercise improves lower extremity muscle strength and delays age-related declines in walking speed and physical function in older adults. Eur. Rev. Aging Phys. Act..

[27] Stuart CA, Brannon MF, Stone WL, Stone MH (2016). Reply to “Letter to the editor: Comments on Stuart et al. (2016): ‘Myosin content of individual human muscle fibers isolated by laser capture microdissection’“. Am. J. Physiol. Cell Physiol..

[28] Mi H (2019). Protocol Update for large-scale genome and gene function analysis with the PANTHER classification system (v.14.0). Nat. Protoc..

[29] Hargreaves M, Spriet LL (2020). Author correction: skeletal muscle energy metabolism during exercise. Nat. Metab..

[30] Pilegaard H, Neufer PD (2004). Transcriptional regulation of pyruvate dehydrogenase kinase 4 in skeletal muscle during and after exercise. Proc. Nutr. Soc..

[31] Herningtyas EH (2008). Branched-chain amino acids and arginine suppress MaFbx/atrogin-1 mRNA expression via mTOR pathway in C2C12 cell line. Biochim. Biophys. Acta.

[32] Ham DJ, Murphy KT, Chee A, Lynch GS, Koopman R (2014). Glycine administration attenuates skeletal muscle wasting in a mouse model of cancer cachexia. Clin. Nutr..

[33] Wang R, Jiao H, Zhao J, Wang X, Lin H (2018). L-Arginine enhances protein synthesis by phosphorylating mTOR (Thr 2446) in a nitric oxide-dependent manner in C2C12 cells. Oxid. Med. Cell Longev..

[34] Wang R (2022). L-Arginine/nitric oxide regulates skeletal muscle development via muscle fibre-specific nitric oxide/mTOR pathway in chickens. Anim. Nutr..

[35] Hall DT, Ma JF, Marco SD, Gallouzi IE (2011). Inducible nitric oxide synthase (iNOS) in muscle wasting syndrome, sarcopenia, and cachexia. Aging (Albany NY).

[36] Filippin LI (2011). Nitric oxide regulates the repair of injured skeletal muscle. Nitric Oxide.

[37] Wright CR, Ward AC, Russell AP (2017). Granulocyte colony-stimulating factor and its potential application for skeletal muscle repair and regeneration. Mediators Inflamm..

[38] Hara M (2011). G-CSF influences mouse skeletal muscle development and regeneration by stimulating myoblast proliferation. J. Exp. Med..

[39] Jurkat-Rott K, Fauler M, Lehmann-Horn F (2006). Ion channels and ion transporters of the transverse tubular system of skeletal muscle. J. Muscle Res. Cell Motil..

[40] Kanehisa M, Goto S (2000). KEGG: kyoto encyclopedia of genes and genomes. Nucleic Acids Res..

[41] De Paepe B, De Bleecker JL (2013). Cytokines and chemokines as regulators of skeletal muscle inflammation: presenting the case of Duchenne muscular dystrophy. Mediators Inflamm..

[42] De Paepe B, Creus KK, Martin JJ, De Bleecker JL (2012). Upregulation of chemokines and their receptors in Duchenne muscular dystrophy: potential for attenuation of myofiber necrosis. Muscle Nerve.

[43] Demoule A (2005). Expression and regulation of CC class chemokines in the dystrophic (mdx) diaphragm. Am. J. Respir. Cell Mol. Biol..

[44] Raffaello A (2010). JunB transcription factor maintains skeletal muscle mass and promotes hypertrophy. J. Cell Biol..

[45] Trenerry MK, Carey KA, Ward AC, Cameron-Smith D (2007). STAT3 signaling is activated in human skeletal muscle following acute resistance exercise. J. Appl. Physiol. (1985).

[46] Sacheck JM (2007). Rapid disuse and denervation atrophy involve transcriptional changes similar to those of muscle wasting during systemic diseases. FASEB J..

[47] Lecker SH (2004). Multiple types of skeletal muscle atrophy involve a common program of changes in gene expression. FASEB J..

[48] Schiaffino S, Mammucari C (2011). Regulation of skeletal muscle growth by the IGF1-Akt/PKB pathway: insights from genetic models. Skelet. Muscle.

[49] Sherman BT (2022). DAVID: a web server for functional enrichment analysis and functional annotation of gene lists (2021 update). Nucleic Acids Res..

[50] Comfort P (2021). Effects of spaceflight on musculoskeletal health: a systematic review and meta-analysis, considerations for interplanetary travel. Sports Med..

[51] Juhl OJ (2021). Update on the effects of microgravity on the musculoskeletal system. NPJ Microgravity.

[52] Lee PHU, Chung M, Ren Z, Mair DB, Kim DH (2022). Factors mediating spaceflight-induced skeletal muscle atrophy. Am. J. Physiol. Cell Physiol..

[53] Ewels P, Magnusson M, Lundin S, Käller M (2016). MultiQC: summarize analysis results for multiple tools and samples in a single report. Bioinformatics.

[54] Dobin A (2013). STAR: ultrafast universal RNA-seq aligner. Bioinformatics.

[55] Dewey CN (2011). Positional orthology: putting genomic evolutionary relationships into context. Brief. Bioinform.

[56] Love MI, Huber W, Anders S (2014). Moderated estimation of fold change and dispersion for RNA-seq data with DESeq2. Genome Biol..

