# Supplementary material for: Human skeletal muscle tissue chip autonomous payload reveals changes in fiber type and metabolic gene expression due to spaceflight
Source: NPJ Microgravity. 2023 Sep 15;9:77. doi: 10.1038/s41526-023-00322-y (PMC10504373; doi:10.1038/s41526-023-00322-y)
Supplement: Supplementary file 1 — Supplementary Material [file 41526_2023_322_MOESM1_ESM.pdf]

Supplementary Material

Human Skeletal Muscle Tissue Chip Autonomous Payload Reveals Changes in Fiber Type and Metabolic Gene Expression Due to Spaceflight

Maddalena Parafati[1*], Shelby Giza[1*], Tushar S. Shenoy[1], Jorge A. Mojica-Santiago[2], Meghan Hopf[3,] Legrand K. Malany[4], Don Platt[5], Isabel Moore[6], Zachary A. Jacobs[6], Paul Kuehl[6], Jason Rexroat[6], Gentry Barnett[6], Christine E. Schmidt[2], William T. McLamb[6], Twyman Clements[6], Paul M. Coen[3] and Siobhan Malany[1].

[1]Department of Pharmacodynamics, College of Pharmacy, University of Florida, Florida 32610, USA.

[2]J. Crayton Pruitt Family Department of Biomedical Engineering, Herbert Wertheim College of Engineering, University of Florida, Gainesville, Florida 32610, USA

[3]Translational Research Institute, AdventHealth, Orlando, Florida 32804, USA

[4]Micro-gRx, INC, Orlando, Florida 32935, USA

[5]Micro Aerospace Solutions, INC, Melbourne, Florida 32935, USA

[6]Space Tango, LLC, Lexington, Kentucky 40505, USA

*Authors contributed equally

This document contains Supplementary Tables 1 through 7 and Figures 1 and 2.

Supplementary Table 1A and B. RNA quantification and RNA Integrity Number (RIN) for (A) flight samples and (B) Ground samples (p. S2)
Supplementary Table 2. Differentially expressed genes in Flight versus Ground young, athletic derived myobundles (pp. S3-7)
Supplementary Table 3. Differentially expressed genes in Flight versus Ground old, sedentary derived myobundles (pp. S8-15)
Supplementary Table 4. Gene Ontology enrichment analysis in Flight versus Ground young, athletic derived myobundles (p. S16)
Supplementary Table 5. Gene Ontology enrichment analysis in Flight versus Ground old, sedentary derived myobundles (pp. S17-18)
Supplementary Table 6. Differentially expressed genes in old, sedentary vs young, athletic derived myobundles in Ground culturing condition (pp. 19-21)
Supplementary Table 7. Differentially expressed genes in old, sedentary vs young, athletic derived myobundles in Flight culturing condition (pp. 22-24)
Supplementary Figure 1. Panther classification system analysis (p.25)
Supplementary Figure 2. Gene ontology (GO) annotation (p. 26)
Supplementary Figure 3. Unique and common DEGs comparing ground OS vs YA and flight OS vs YA datasets (pp. 27-28).

Supplementary Table 1. RNA quantification and RNA Integrity Number (RIN) for (A) flight samples and (B) Ground samples.

A. RNA quantity for Flight samples

Chip #	Description	RNA (ng)	RIN
1	Old Estim	1637.7	8.7
2	Old Estim	1216.7	7.1
3	Old Estim	1139.3	7.2
4	Young Estim	1497.8	7.2
5	Young Estim	1434.4	6.8
6	Young Estim	1725.7	9.6
7	Young No Estim	1312.2	7.2
8	Young Estim	1821.8	9.1
9	Old No Estim	1753.2	6.9
10	Old No Estim	2225.6	7.0*
11	Old No Estim	1007.8	7.1*
12	Old No Estim	3505.4	9.0*
13	Young No Estim	1878.7	8.9*
14	Young No Estim	884.1	6.2
15	Young No Estim	1710	7.2*
16	Young No Estim	1188.6	9.0*

B. RNA quantity for Ground samples

Chip #	Description	RNA (ng)	RIN
1	Old No Estim	1395	9.3*
2	Old No Estim	1014	9.3*
3	Old No Estim	1234	9.2*
4	Old No Estim	1650	9.5
5	Young No Estim	1500	9.1*
6	Young No Estim	990	9.1*
7	Young No Estim	1456	9.3*
8	Young No Estim	1284	9.0

*Tissue chips used in the analysis and reported in the communication. Each chip is derived from cells pooled from 5 male donors of the YA cohort or the OS cohort. Estim and No Estim indicates tissue chips that were stimulated over the time course of the experiment vs tissue chips that were not electrically stimulated, respectively.

Supplementary Table 2. Differentially expressed genes in Flight versus Ground young, athletic derived myobundles

Gene	Description	Fold Change	FDR
	**Upregulated Genes (44)**		
AC093525.2	AC093525.2 readthrough	7.164650421	5.21275E-05
CNTNAP2	contactin associated protein 2	5.450281716	0.000617734
GRIA4	glutamate ionotropic receptor AMPA type subunit 4	5.366105495	4.16413E-05
CSF3R	colony stimulating factor 3 receptor	5.048318314	0.040861248
OR51E2	olfactory receptor family 51 subfamily E member 2	4.982648803	0.000195307
DYDC2	DPY30 domain containing 2	4.868519969	0.032258577
CPB1	carboxypeptidase B1	4.157978745	0.025455092
METTL21C	methyltransferase 21C, AARS1 lysine	3.814856526	0.0009246
MCHR1	melanin concentrating hormone receptor 1	3.135941902	2.71353E-07
UMODL1	uromodulin like 1	2.992139178	0.024107585
AC018630.2	AC018630.2 readthrough	2.838326714	0.031842359
ABCG4	ATP binding cassette subfamily G member 4	2.768271531	0.024622362
KCNJ4	potassium inwardly rectifying channel subfamily J member 4	2.597140863	0.000184233
MYRFL	myelin regulatory factor like	2.574460737	1.93839E-06
VWDE	von Willebrand factor D and EGF domains	2.547118719	0.005281277
PFKP	phosphofructokinase, platelet	2.544060278	4.67614E-06
HS6ST3	heparan sulfate 6-O-sulfotransferase 3	2.511162382	0.023621889
COL6A6	collagen type VI alpha 6 chain	2.458016866	0.004006578
TGFBR3L	transforming growth factor beta receptor 3 like	2.42910639	0.024866346
MDFI	MyoD family inhibitor	2.38453661	0.043843058
SERPINA5	serpin family A member 5	2.369500506	0.003012647
PODNL1	podocan like 1	2.324977308	3.5276E-14
FGF12	fibroblast growth factor 12	2.295016809	0.017954825
SLC6A1	solute carrier family 6 member 1	2.287422637	0.007291892
MGP	matrix Gla protein	2.272479259	0.001483125
SCIN	scinderin	2.260979666	0.001273009
ZNF850	zinc figner protein 850	2.251668785	0.002335027
SLCO4A1	solute carrier organic anion transporter family member	2.249036509	1.16401E-07
B3GNT4	UDP-GlcNAc:betaGal beta-1,3-N-acetylglucosaminyltransferase 4	2.244322193	0.028133397

GRM1	glutamate metabotropic receptor 1	2.231068317	3.6986E-05
TTC34	tetratricopeptide repeat domain 34	2.186841093	0.016333667
FAM178B	FAM178B: family with sequence similarity 178 member B	2.183341716	0.001413665
FAM57B	FAM57B: family with sequence similarity 57 member B	2.156290814	7.54584E-15
PALM3	paralemmin 3	2.140786839	0.032658474
TOR4A	torsin family 4 member A	2.127319267	1.89407E-10
YPEL4	yipee like 4	2.121103069	0.028785587
CTRL	chymotrypsin like	2.113328046	0.01915699
PLB1	phospholipase B1	2.113285066	0.023783155
CHAC1	ChaC glutathione specific gamma-glutamylcyclotransferase 1	2.113091053	0.001268902
DCDC1	doublecortin domain containing 1	2.101303893	0.029020203
PNLIPRP3	pancreatic lipase related protein 3	2.070574498	0.021410201
C7	complement C7	2.067699643	7.46116E-07
SUSD4	sushi domain containing 4	2.036046359	1.60688E-17
VIL1	villin 1	2.00816373	0.04739108
	**Downregulated Genes (110)**		
GUCY1A2	guanylate cyclase 1 soluble subunit alpha 2	-2.018093048	1.99411E-10
ZFP36	ZFP36 ring finger protein	-2.018876449	3.01373E-06
EFEMP1	EGF containing fibulin extracellular matrix protein 1	-2.027015633	0.000259005
RIMS3	regulating synaptic membrane exocytosis 3	-2.033630867	0.005667261
PCDHA7	protocadherin alpha 7	-2.034751645	0.028353136
PDE11A	phosphodiesterase 11A	-2.043430345	0.037300533
TMEM178B	transmembrane protein 178B	-2.043941926	5.64787E-36
EGR2	early growth response 2	-2.049376215	6.8619E-05
SPAAR	small regulatory polypeptide of amino acid response	-2.057438265	0.026079467
BIRC3	baculoviral IAP repeat containing 3	-2.065881453	8.43477E-06
NGFR	nerve growth factor receptor	-2.071120425	6.1174E-05
PHACTR3	phosphatase and actin regulator 3	-2.076778696	0.000204077
OAS3	2'-5'-oligoadenylate synthetase 3	-2.087824987	0.000469052
CXCL8	C-X-C motif chemokine ligand 8	-2.125016841	0.016734854
SHISA9	shisa family member 9	-2.126294751	4.22057E-09
JUNB	JunB proto-oncogene, AP-1 transcription factor subunit	-2.127731456	0.000641766
ITGB3	integrin subunit beta 3	-2.131777766	6.56226E-08
FAM43B	family with sequence similarity 43 member B	-2.13361598	0.033720356
CDHR1	cadherin related family member 1	-2.134569371	6.77655E-05

ALS2CR12	ALS2CR12 readthrough	-2.142778176	0.00123712
C11orf96	chromosome 11 open reading frame 96	-2.148358618	0.000238374
CLIC2	chloride intracellular channel 2	-2.156646387	0.036441058
PCDH10	protocadherin 10	-2.162674866	0.00519119
PTX3	pentraxin 3	-2.169064812	3.50516E-05
RCCD1	RCC1 domain containing 1	-2.200601598	0.028435238
AC073896.1	AC073896.1 readthrough	-2.200991483	0.000140442
GCNT1	glucosaminyl (N-acetyl) transferase 1	-2.243308128	0.027705945
GIPC2	GIPC PDZ domain containing family member 2	-2.269410671	0.012464612
TLE2	TLE family member 2, transcriptional corepressor	-2.269757409	0.015884135
OTUD1	OTU deubiquitinase 1	-2.287705143	6.79091E-15
MYH6	myosin heavy chain 6	-2.290182333	8.65344E-07
SBK2	SH3 domain binding kinase family member 2	-2.294608569	0.013172591
CORO2A	coronin 2A	-2.300680372	0.024373908
RANBP17	RAN binding protein 17	-2.314937945	0.040476689
GNRHR	gonadotropin releasing hormone receptor	-2.325688078	0.00815339
C8orf34	chromosome 8 open reading frame 34	-2.33723793	0.028258487
KCNA10	potassium voltage-gated channel subfamily A member 10	-2.343994096	0.02806624
IGSF3	immunoglobulin superfamily member 3	-2.355276695	5.4917E-05
MINOS1-NBL1	MICOS10-NBL1 readthrough	-2.389441362	0.000278839
CSF3	colony stimulating factor 3	-2.438831523	5.61648E-05
CA7	carbonic anhydrase 7	-2.446927221	7.81941E-06
ADGRD1	adhesion G protein-coupled receptor D1	-2.450835201	8.09285E-12
ITIH3	inter-alpha-trypsin inhibitor heavy chain 3	-2.457215484	0.022896877
CTXN3	cortexin 3	-2.459783114	3.56648E-14
EGR1	early growth response 1	-2.485448008	0.000102019
SLC29A4	solute carrier family 29 member 4	-2.501427569	2.20492E-10
ABCA4	ATP binding cassette subfamily A member 4	-2.503758261	0.000635994
TMEM40	transmembrane protein 40	-2.551979434	0.004537744
CDYL2	chromodomain Y like 2	-2.554687625	2.66455E-18
MGAT4C	MGAT4 family member C	-2.557487954	0.033624859
CD24	CD24 molecule	-2.590494325	5.89464E-06
GABRB3	gamma-aminobutyric acid type A receptor subunit beta3	-2.60096351	0.000777337
ADAMTSL4	ADAMTS like 4	-2.621886446	4.6055E-26
DNAH2	dynein axonemal heavy chain 2	-2.625782585	0.000620396

SCN3A	sodium voltage-gated channel alpha subunit 3	-2.634804574	0.000207852
CNTN3	contactin 3	-2.66779935	4.33268E-05
MAS1	MAS1 proto-oncogene, G protein-coupled receptor	-2.694376738	8.96184E-09
PRDM6	PR/SET domain 6	-2.700197512	0.010559536
OR7A5	olfactory receptor family 7 subfamily A member 5	-2.70300847	0.008383645
MTUS2	microtubule associated scaffold protein 2	-2.706234786	1.02358E-05
SLCO2B1	solute carrier organic anion transporter family member 2B1	-2.71005521	0.019104909
CUX2	cut like homeobox 2	-2.715096495	0.012893493
SCN7A	sodium voltage-gated channel alpha subunit 7	-2.72408044	0.013424219
CXCL5	C-X-C motif chemokine ligand 5	-2.779740703	0.017285992
VMO1	vitelline membrane outer layer 1 homolog	-2.798735883	0.016978677
SOCS1	suppressor of cytokine signaling 1	-2.811750603	0.004700644
MMP9	matrix metallopeptidase 9	-2.826698247	0.045205873
FAM19A2	FAM19A2 readthrough	-2.838004149	0.014019371
CACNA1E	calcium voltage-gated channel subunit alpha1 E	-2.858147793	0.005064968
ONECUT2	one cut homeobox 2	-2.915792192	0.00048654
INSC	INSC spindle orientation adaptor protein	-2.916515798	0.014456608
DGAT2	diacylglycerol O-acyltransferase 2	-2.919201125	0.045426703
DMBT1	deleted in malignant brain tumors 1	-2.94457787	2.44486E-05
FOS	Fos proto-oncogene, AP-1 transcription factor subunit	-2.991999455	0.000247171
CXCL13	C-X-C motif chemokine ligand 13	-3.067756845	3.47741E-16
ADRB2	adrenoceptor beta 2	-3.096994028	7.76539E-19
SERPINB7	serpin family B member 7	-3.187850298	0.00519119
FLRT1	fibronectin leucine rich transmembrane protein 1	-3.270832353	0.019496782
PTPRC	protein tyrosine phosphatase receptor type C	-3.287150062	0.006176527
RELN	reelin	-3.319118652	0.000292176
PDK4	pyruvate dehydrogenase kinase 4	-3.325146285	3.85134E-05
FOSB	FosB proto-oncogene, AP-1 transcription factor subunit	-3.375890553	7.71904E-07
ANO2	anoctamin 2	-3.446938388	0.003711354
ESM1	endothelial cell specific molecule 1	-3.500513259	0.007727148
KISS1	KiSS-1 metastasis suppressor	-3.600023239	0.0001874
SSTR2	somatostatin receptor 2	-3.619293532	0.009280342
TMEM110-MUSTN1	TMEM110-MUSTN1 readthrough	-3.648751254	7.35509E-05
SMTNL1	smoothelin like 1	-3.6934315	1.00875E-06
MYL10	myosin light chain 10	-3.95865901	9.39304E-08

PDZD2	PDZ domain containing 2	-3.98994048	0.00136236
SCARA5	scavenger receptor class A member 5	-4.009235804	7.62091E-21
PVALB	parvalbumin	-4.0198962	0.000257454
SYT1	synaptotagmin 1	-4.034078439	0.000140442
PSG11	pregnancy specific beta-1-glycoprotein 11	-4.113972439	0.012018776
CUZD1	CUB and zona pellucida like domains 1	-4.116254828	1.61224E-05
DIRAS2	DIRAS family GTPase 2	-4.118507424	0.007374237
TM4SF20	transmembrane 4 L six family member 20	-4.166515956	0.000391648
DEFB103B	defensin beta 103B	-4.214503417	0.000139906
KCNA2	potassium voltage-gated channel subfamily A member 2	-4.217102228	0.006392639
AC013489.1	AC013489.1 readthrough	-4.29376469	0.032170351
STEAP4	STEAP4 metalloreductase	-4.403227272	0.002524526
RPTN	repetin	-4.470907609	8.15675E-06
KPRP	keratinocyte proline rich protein	-4.526968504	0.000157524
LY75-CD302	LY75-CD302 readthrough	-4.591585426	0.027003205
CXCL6	C-X-C motif chemokine ligand 6	-4.602979064	0.006890469
CISH	cytokine inducible SH2 containing protein	-4.645653524	0.001169639
GCNT3	glucosaminyl (N-acetyl) transferase 3, mucin type	-4.665883943	3.33171E-15
SERPINB2	serpin family B member 2	-5.469421134	0.001240009
SULT1A4	sulfotransferase family 1A member 4	-5.943844153	0.007648353
PSG1	pregnancy specific beta-1-glycoprotein 1	-6.233947331	0.001443649

Supplementary Table 3. Differentially expressed genes in Flight versus Ground old, sedentary derived myobundles

Gene	Description	Fold Change	FDR
	**Upregulated Genes (99)**		
HIST2H2AA3	HIST2H2AA3 readthrough	9.996972635	0.014365614
AC048338.1	AC048338.1 readthrough	9.896124868	6.53643E-06
CSF3R	colony stimulating factor 3 receptor	7.336449293	0.000980146
HBA2	hemoglobin subunit alpha 2	6.629040764	0.005256727
AP000350.4	AP000350.4 readthrough	6.200975095	0.008942407
SLC14A1	solute carrier family 14 member 1 (Kidd blood group)	5.833977572	0.001174276
KRT19	keratin 19	5.284363775	0.00127304
RSAD2	radical S-adenosyl methionine domain containing 2	5.057491019	0.000376949
GAL3ST1	galactose-3-O-sulfotransferase 1	4.440323571	0.000592674
CPB1	carboxypeptidase B1	4.384920788	0.000258081
FSCN3	fascin actin-bundling protein 3	4.193087119	0.000633467
KRT8	keratin 8	4.151324259	0.015616573
ENO4	enolase 4	4.085678152	0.008847841
METTL21C	methyltransferase 21C, AARS1 lysine	3.965837441	0.000487923
ARHGAP36	Rho GTPase activating protein 36	3.916108217	0.030072269
RORC	RAR related orphan receptor C	3.893111603	4.48508E-38
KCNJ3	potassium inwardly rectifying channel subfamily J member 3	3.784502622	0.00083577
ST20-MTHFS	ST20-MTHFS readthrough	3.762067682	0.001953065
NOS1	nitric oxide synthase 1	3.703729233	3.58378E-15
FAM57B	FAM57B readthrough	3.703378318	9.72522E-45
SERPINA5	serpin family A member 5	3.679110525	2.27285E-07
LCP1	lymphocyte cytosolic protein 1	3.671761138	0.011029286
CNTNAP2	contactin associated protein 2	3.538737469	0.003434529
OR51E2	olfactory receptor family 51 subfamily E member 2	3.44537276	0.000345925
IRF4	interferon regulatory factor 4	3.417250424	0.013594084
HSD11B2	hydroxysteroid 11-beta dehydrogenase 2	3.26539635	5.30574E-09
FAM83E	family with sequence similarity 83 member E	3.187961906	0.044733397
MTCP1	mature T cell proliferation 1	3.178835087	0.014035233
LYZ	lysozyme	3.149502048	0.002253136
SLCO4A1	solute carrier organic anion transporter family member 4A1	3.132334346	2.38179E-14
MYRFL	myelin regulatory factor like	3.081932779	6.03269E-09

CH25H	cholesterol 25-hydroxylase	3.07467968	0.006172311
ADAM8	ADAM metallopeptidase domain 8	3.026603305	0.001069424
CSKMT	citrate synthase lysine methyltransferase	2.984105134	1.48198E-08
KIAA0040	KIAA0040 readthrough	2.897171856	0.000153288
RAB33A	RAB33A, member RAS oncogene family	2.880591264	0.020934461
CORO1A	coronin 1A	2.879991295	0.039013179
SLC37A2	solute carrier family 37 member 2	2.876235031	0.031833815
TRIB3	tribbles pseudokinase 3	2.872474462	8.90624E-18
LLGL2	LLGL scribble cell polarity complex component 2	2.844440683	1.44856E-09
SLC6A9	solute carrier family 6 member 9	2.820442933	2.7412E-05
C2CD4A	C2 calcium dependent domain containing 4A	2.802341488	1.80206E-08
CHAD	chondroadherin	2.793136991	0.002692199
CHAC1	ChaC glutathione specific gamma-glutamylcyclotransferase 1	2.791954301	1.58653E-06
LINGO4	leucine rich repeat and Ig domain containing 4	2.77428638	0.02268445
NPBWR1	neuropeptides B and W receptor 1	2.770595739	0.000171063
SCUBE2	signal peptide, CUB domain and EGF like domain containing 2	2.725747424	1.63132E-21
C4orf17	chromosome 4 open reading frame 17	2.715711659	0.030599871
PCK2	phosphoenolpyruvate carboxykinase 2, mitochondrial	2.688584044	2.39918E-19
MX1	MX dynamin like GTPase 1	2.627265865	0.000312272
SLC9A3	solute carrier family 9 member A3	2.624480958	0.00451659
ABCB4	ATP binding cassette subfamily B member 4	2.613250985	0.022695037
CA14	carbonic anhydrase 14	2.600229786	0.00088289
PCLAF	PCNA clamp associated factor	2.600007731	0.013165177
PHGDH	phosphoglycerate dehydrogenase	2.597687813	9.60059E-16
PLCB2	phospholipase C beta 2	2.587428562	5.05261E-05
KRT18	keratin 18	2.556450727	1.31829E-05
ADRA2A	adrenoceptor alpha 2A	2.554008547	7.19851E-13
SLC6A1	solute carrier family 6 member 1	2.545584324	0.001193861
GCOM1	GCOM1, MYZAP-POLR2M combined locus	2.543365656	0.009504753
APLN	apelin	2.485238218	0.003614168
MMP9	matrix metallopeptidase 9	2.480190644	0.041806699
GRM1	glutamate metabotropic receptor 1	2.451745383	5.76266E-07
ELN	elastin	2.442668494	1.42308E-18
TRPM8	transient receptor potential cation channel subfamily M member 8	2.43841871	1.609E-05
KCNRG	potassium channel regulator	2.424743962	0.005477797

PLIN4	perilipin 4	2.418158315	2.50827E-15
ABCG1	ATP binding cassette subfamily G member 1	2.409928463	9.60356E-26
ASB10	ankyrin repeat and SOCS box containing 10	2.409437385	2.6281E-06
PERM1	PPARGC1 and ESRR induced regulator, muscle 1	2.393578989	3.37929E-09
GLDN	gliomedin	2.390485559	4.17275E-05
BRICD5	BRICHOS domain containing 5	2.37946023	0.017334931
OAS1	2'-5'-oligoadenylate synthetase 1	2.358644057	0.017324701
NDRG4	NDRG family member 4	2.348510796	2.53496E-12
IFITM1	interferon induced transmembrane protein 1	2.341173199	0.000385455
TFCP2L1	transcription factor CP2 like 1	2.330740277	0.043211995
FHAD1	forkhead associated phosphopeptide binding domain 1	2.310468721	0.003984946
NOXO1	NADPH oxidase organizer 1	2.303047422	0.000168865
FAM178B	family with sequence similarity 178 member B	2.296591338	0.000829266
THRSP	thyroid hormone responsive	2.291401212	0.004577044
MCHR1	melanin concentrating hormone receptor 1	2.280855022	0.00021079
EXOC3L1	exocyst complex component 3 like 1	2.237784772	1.18055E-05
POU2F2	POU class 2 homeobox 2	2.225137741	0.038942049
NRN1L	neuritin 1 like	2.208437259	0.000551956
ARG2	arginase 2	2.181974414	6.02253E-07
HSPA2	heat shock protein family A (Hsp70) member 2	2.181613481	0.001367034
VIL1	villin 1	2.154467686	0.027073905
RTN4RL2	reticulon 4 receptor like 2	2.143646973	6.39305E-10
EGFL7	EGF like domain multiple 7	2.130354194	7.92453E-25
RASGRP2	RAS guanyl releasing protein 2	2.105147255	1.17602E-10
PFKP	phosphofructokinase, platelet	2.079083902	0.000185431
CD101	CD101 molecule	2.049526837	3.26453E-05
FAM129A	FAM129A readthrough	2.037754959	1.02031E-21
KCNS3	potassium voltage-gated channel modifier subfamily S member 3	2.026461785	5.67255E-14
TOR4A	torsin family 4 member A	2.02357543	5.99937E-11
CCDC28B	coiled-coil domain containing 28B	2.020495327	1.16227E-13
PRODH	proline dehydrogenase 1	2.020243469	4.24532E-11
COL15A1	collagen type XV alpha 1 chain	2.014387784	8.45469E-18
ADM2	adrenomedullin 2	2.013952235	0.004454598
CBS	cystathionine beta-synthase	2.002847428	4.34413E-13
	**Downregulated Genes (153)**		

PAPPA	pappalysin 1	-2.001698621	1.68884E-05
MMP19	matrix metallopeptidase 19	-2.004168809	0.00024325
CAPN9	calpain 9	-2.00701002	0.001289031
ID1	inhibitor of DNA binding 1	-2.008436028	0.001189951
EFEMP1	EGF containing fibulin extracellular matrix protein 1	-2.01222761	8.39305E-05
SERPINE1	serpin family E member 1	-2.012798599	0.007341419
MID1	midline 1	-2.014133958	2.71322E-08
ADGRV1	adhesion G protein-coupled receptor V1	-2.016315779	8.46852E-06
NCALD	neurocalcin delta	-2.024698413	3.27892E-13
MAPK4	mitogen-activated protein kinase 4	-2.031338479	5.82168E-08
PLCXD2	phosphatidylinositol specific phospholipase C X domain containing 2	-2.048349705	0.001047584
MINAR1	membrane integral NOTCH2 associated receptor 1	-2.052158468	1.47777E-18
DLK1	delta like non-canonical Notch ligand 1	-2.06513025	0.008519671
SSC5D	scavenger receptor cysteine rich family member with 5 domains	-2.065761861	5.89858E-06
L3MBTL4	L3MBTL histone methyl-lysine binding protein 4	-2.075094071	0.016297521
EXPH5	exophilin 5	-2.088316686	0.001693647
NKD1	NKD inhibitor of WNT signaling pathway 1	-2.089314247	4.99209E-06
CNTN3	contactin 3	-2.101849509	0.001064981
MAP6	microtubule associated protein 6	-2.105741108	0.000477822
PALM2	paralemmin 2	-2.106223896	1.2873E-10
PLGLB1	plasminogen like B1	-2.110172055	0.023820057
CPM	carboxypeptidase M	-2.118777225	0.018017253
OTUD1	OTU deubiquitinase 1	-2.130851869	1.51378E-13
FAM184B	family with sequence similarity 184 member B	-2.132789747	5.04975E-07
CHIT1	chitinase 1	-2.143020897	0.000140463
ADRB2	adrenoceptor beta 2	-2.152287789	2.9653E-10
PAMR1	peptidase domain containing associated with muscle regeneration 1	-2.168966168	5.07082E-10
CMTM5	CKLF like MARVEL transmembrane domain containing 5	-2.181187893	0.000343903
GUCY1A2	guanylate cyclase 1 soluble subunit alpha 2	-2.182501382	1.84819E-14
CXCL5	C-X-C motif chemokine ligand 5	-2.185653502	0.043003003
PRR18	proline rich 18	-2.187533016	0.01263233
SAMD13	sterile alpha motif domain containing 13	-2.195411319	0.014011783
SLC6A17	solute carrier family 6 member 17	-2.199972562	1.59345E-05
PPP1R1A	protein phosphatase 1 regulatory inhibitor subunit 1A	-2.203083641	8.02612E-06
MFAP5	microfibril associated protein 5	-2.20389193	3.08199E-05

POU5F1B	POU class 5 homeobox 1B	-2.20902513	1.19406E-07
ADAMTSL4	ADAMTS like 4	-2.212038108	2.71408E-19
C19orf81	chromosome 19 open reading frame 81	-2.223905987	0.032471623
RNF165	ring finger protein 165	-2.226441009	0.045548378
AGXT	alanine--glyoxylate aminotransferase	-2.227146998	0.011745612
KCNE5	potassium voltage-gated channel subfamily E regulatory subunit 5	-2.232802059	1.68087E-09
ACTN3	actinin alpha 3	-2.236539699	1.94493E-05
LGI1	leucine rich glioma inactivated 1	-2.244541331	0.00129478
PCDHB3	protocadherin beta 3	-2.248507738	0.041636754
PPP1R27	protein phosphatase 1 regulatory subunit 27	-2.250189692	2.95039E-07
P2RX1	purinergic receptor P2X 1	-2.256160133	9.65365E-06
TMEM217	transmembrane protein 217	-2.265371971	0.043131825
C8orf34	chromosome 8 open reading frame 34	-2.267123331	0.020709145
SERPINB7	serpin family B member 7	-2.267475774	0.031491506
SPAG6	sperm associated antigen 6	-2.271276579	0.000294406
CDH8	cadherin 8	-2.275398912	1.76668E-07
MGAM	maltase-glucoamylase	-2.289340221	0.002179971
AC008073.3	AC008073.3 readthrough	-2.29185413	0.023138575
SLC38A4	solute carrier family 38 member 4	-2.29421038	0.004517197
CPXM2	carboxypeptidase X, M14 family member 2	-2.295883371	0.036290694
CDK5R2	cyclin dependent kinase 5 regulatory subunit 2	-2.299826607	0.016450514
AMPH	amphiphysin	-2.306415442	0.000281947
CDHR1	cadherin related family member 1	-2.307338568	1.08457E-05
CDH22	cadherin 22	-2.316198091	0.025658864
ECM2	extracellular matrix protein 2	-2.319226085	0.000944036
OCA2	OCA2 melanosomal transmembrane protein	-2.320966428	0.046970969
MMP13	matrix metallopeptidase 13	-2.322828809	0.001946904
PLEKHG7	pleckstrin homology and RhoGEF domain containing G7	-2.328091062	0.019518382
PIP5K1B	phosphatidylinositol-4-phosphate 5-kinase type 1 beta	-2.32866678	1.58839E-09
ITGA9	integrin subunit alpha 9	-2.330332409	3.17321E-05
ILDR2	immunoglobulin like domain containing receptor 2	-2.345924597	0.005777283
SCN2A	sodium voltage-gated channel alpha subunit 2	-2.351689839	0.01612589
IL32	interleukin 32	-2.356595964	0.00101918
HSD11B1	hydroxysteroid 11-beta dehydrogenase 1	-2.362218595	0.000616679
NPAS1	neuronal PAS domain protein 1	-2.38509115	2.14913E-09
CELF4	CUGBP Elav-like family member 4	-2.397723746	0.01864044

SDK2	sidekick cell adhesion molecule 2	-2.414618146	5.00587E-30
FAM43B	family with sequence similarity 43 member B	-2.417441818	0.005734886
RELN	reelin	-2.443817102	0.003483523
BTBD8	BTB domain containing 8	-2.475392377	0.000984211
LONRF2	LON peptidase N-terminal domain and ring finger 2	-2.478477798	2.16136E-11
SCN7A	sodium voltage-gated channel alpha subunit 7	-2.482115699	0.005655595
ADAP1	ArfGAP with dual PH domains 1	-2.490141884	0.000516603
AIRE	autoimmune regulator	-2.495767873	1.89836E-06
DPYSL5	dihydropyrimidinase like 5	-2.509677934	1.68175E-15
NHLH2	nescient helix-loop-helix 2	-2.515217017	9.32663E-30
GNAT1	G protein subunit alpha transducin 1	-2.526506819	0.029138282
UGT2B4	UDP glucuronosyltransferase family 2 member B4	-2.571625403	3.85685E-06
TMEM178B	transmembrane protein 178B	-2.59172763	1.17119E-59
GJA5	gap junction protein alpha 5	-2.609205441	1.64855E-08
AFF3	ALF transcription elongation factor 3	-2.652820225	0.047420947
MYH6	myosin heavy chain 6	-2.660505638	3.32316E-09
MYH1	myosin heavy chain 1	-2.667226287	4.28409E-13
INA	internexin neuronal intermediate filament protein alpha	-2.668632236	0.010260118
NKAIN4	sodium/potassium transporting ATPase interacting 4	-2.683826755	0.034810829
DCX	doublecortin	-2.709482013	3.53112E-08
DIRAS2	DIRAS family GTPase 2	-2.731609283	0.0080137
BEGAIN	brain enriched guanylate kinase associated	-2.737767166	0.037039297
ZNF781	zinc finger protein 781	-2.750962443	0.041433385
SCARA5	scavenger receptor class A member 5	-2.768009597	4.39118E-10
GALR1	galanin receptor 1	-2.801132683	0.037139691
PDK4	pyruvate dehydrogenase kinase 4	-2.831019397	0.00051737
PDZD2	PDZ domain containing 2	-2.850566244	0.010004297
NGFR	nerve growth factor receptor	-2.879977093	3.16514E-13
NRARP	NOTCH regulated ankyrin repeat protein	-2.967003151	0.034104813
RPTN	repetin	-2.975318581	0.00362023
AC010422.3	AC010422.3 readthrough	-3.034696001	0.033209818
ARC	activity regulated cytoskeleton associated protein	-3.043765555	0.001840919
GDF6	growth differentiation factor 6	-3.04822861	9.08586E-15
SAMD15	sterile alpha motif domain containing 15	-3.05838817	0.023021402
C12orf42	chromosome 12 open reading frame 42	-3.098787019	0.005038486
TBC1D30	TBC1 domain family member 30	-3.116741964	1.85836E-06

C1QL4	complement C1q like 4	-3.122095899	0.005801968
RYR3	ryanodine receptor 3	-3.152862339	6.23527E-06
CACNA1E	calcium voltage-gated channel subunit alpha1 E	-3.154784862	0.001024993
KCNQ1	potassium voltage-gated channel subfamily Q member 1	-3.172358287	1.70395E-07
GSG1	germ cell associated 1	-3.173827976	5.15447E-05
ESRP1	epithelial splicing regulatory protein 1	-3.184071355	0.02214051
GPBAR1	G protein-coupled bile acid receptor 1	-3.196218517	0.044440742
IGF1	insulin like growth factor 1	-3.212495355	0.007945092
FREM2	FRAS1 related extracellular matrix 2	-3.263565817	2.07025E-54
LRRIQ1	leucine rich repeats and IQ motif containing 1	-3.297080775	0.010536406
CD24	CD24 molecule	-3.306524939	1.5196E-09
RASSF2	Ras association domain family member 2	-3.341046201	3.70519E-06
ZNF730	zinc finger protein 730	-3.355318377	0.031543199
PSG11	pregnancy specific beta-1-glycoprotein 11	-3.396968389	0.022314738
DRP2	dystrophin related protein 2	-3.39969905	4.87849E-36
CCDC170	coiled-coil domain containing 170	-3.419722597	0.0184137
STMN2	stathmin 2	-3.438255383	7.39406E-11
CUX2	cut like homeobox 2	-3.474253	0.00101918
MYH2	myosin heavy chain 2	-3.493656227	6.68099E-24
CXCL13	C-X-C motif chemokine ligand 13	-3.680230955	4.13211E-20
MALL	mal, T cell differentiation protein like	-3.684467646	1.07442E-08
NTF3	neurotrophin 3	-3.705363066	0.026628825
KPRP	keratinocyte proline rich protein	-3.718420971	3.33559E-05
SCN3A	sodium voltage-gated channel alpha subunit 3	-3.764869992	1.88268E-08
GCNT3	glucosaminyl (N-acetyl) transferase 3, mucin type	-3.826071587	1.63143E-10
SERPINB2	serpin family B member 2	-3.917210704	0.001438626
SERPINB12	serpin family B member 12	-3.936814001	0.000239984
CSMD1	CUB and Sushi multiple domains 1	-3.94524382	1.15316E-09
ADGRD1	adhesion G protein-coupled receptor D1	-3.968823097	6.57796E-28
ADGRB3	adhesion G protein-coupled receptor B3	-3.98337514	0.004901084
NLRP10	NLR family pyrin domain containing 10	-4.157116629	0.005115268
INSC	INSC spindle orientation adaptor protein	-4.194969484	0.001832198
MAS1	MAS1 proto-oncogene, G protein-coupled receptor	-4.374162588	6.0226E-22
CHRM2	cholinergic receptor muscarinic 2	-4.403697891	0.017115536
MYL10	myosin light chain 10	-4.419396312	1.17555E-07
KISS1	KiSS-1 metastasis suppressor	-4.423496137	2.69415E-07

CTXN3	cortexin 3	-4.507775368	2.69496E-35
KCNA10	potassium voltage-gated channel subfamily A member 10	-4.584129996	1.61719E-05
SAA1	serum amyloid A1	-4.86295248	0.021436073
TM4SF20	transmembrane 4 L six family member 20	-5.05040186	0.000332029
SLC5A1	solute carrier family 5 member 1	-5.151840374	0.004103828
AL049839.2	AL049839.2 readthrough	-5.545299993	0.040980606
AC018512.1	AC018512.1 readthrough	-5.712811369	0.003424014
PVALB	parvalbumin	-6.963866832	2.01617E-05
CEMP1	cementum protein 1	-7.01121302	0.034021725
DEFB103B	defensin beta 103B	-7.244418203	3.71178E-07

Supplementary Table 4. Gene Ontology enrichment analysis in Flight versus Ground young, athletic derived myobundle

GO term categories	Term	Count	p-value	Genes
Cellular Component	GO:0016021; integral component of plasma membrane	51	0.0043	*GABRB3, CNTNAP2, CSF3R, STEAP4, MYRFL, SLC6A1, CXCL13, PLB1, GRM1, HS6ST3, SLCO2B1, GNRHR, DGAT2, TMEM178B, TMEM40, SHISA9, ANO2, SLCO4A1, CDHR1, OAS3, B3GNT4, OR7A5, SLC29A4, KCNA10, CTXN3, ABCG4, TOR4A, IGSF3, PCDH10, KCNA2, GCNT1, ADRB2, MCHR1, CUZD1, TGFBR3L, FLRT1, SUSD4, TM4SF20, GCNT3, SCN3A, GRIA4, KCNJ4, NGFR, SYT1, OR51E2, ABCA4, MGAT4C, MAS1, ADGRD1, PTPRC, UMODL1.*
	GO:0005886; plasma membrane	48	0.014	*GABRB3, STEAP4, CSF3R, PCDH10, ITGB3, PALM3, KCNA2, DIRAS2, ADRB2, SLC6A1, CACNA1E, MCHR1, PLB1, GRM1, RELN, PDZD2, SLCO2B1, FLRT1, INSC, TM4SF20, GNRHR, CLIC2, SCN3A, PCDHA7, GRIA4, KCNJ4, NGFR, SERPINB2, CISH, SYT1, SCARA5, OR51E2, PSG1, SSTR2, ANO2, VIL1, MAS1, ADGRD1, PTPRC, SLCO4A1, OAS3, CDHR1, OR7A5, SLC29A4, CNTN3, KCNA10, RCCD1, ABCG4.*
	GO:0016020; membrane	37	0.012	*TOR4A, CNTNAP2, STEAP4, IGSF3, KCNA2, GCNT1, DIRAS2, ADRB2, SLC6A1, CUZD1, HS6ST3, SERPINA5, TGFBR3L, RIMS3, DMBT1, SLCO2B1, SUSD4, TM4SF20, GNRHR, GCNT3, CLIC2, NGFR, DGAT2, TMEM178B, ABCA4, TMEM40, FOS, ADGRD1, PTPRC, OAS3, CDHR1, OR7A5, KCNA10, CTXN3, CD24, ABCG4, PFKP.*
	GO:0005576; extracellular region	30	0.00012	*TOR4A, DEFB103B, CXCL6, CSF3, ITIH3, CSF3R, CXCL8, PSG11, CXCL13, CXCL5, SERPINA5, ESM1, RELN, EFEMP1, PDZD2, C7, DMBT1, ADAMTSL4, SUSD4, RPTN, NGFR, SERPINB2, PSG1, VWDE, MMP9, KISS1, MGP, CNTN3, COL6A6, PTX3.*
	GO:0005615; extracellular space	28	0.00015	*DEFB103B, CXCL6, CSF3, CPB1, CXCL8, GCNT1, CXCL13, CXCL5, SERPINA5, VMO1, TGFBR3L, PODNL1, RELN, EFEMP1, C7, DMBT1, FLRT1, TLE2, SERPINB2, SERPINB7, MMP9, KISS1, CTRL, OAS3, PTX3, FGF12, PNLIPRP3, UMODL1.*
	GO:0005887; integral component of plasma membrane	22	0.00043	*GABRB3, NGFR, CSF3R, SCARA5, PCDH10, ITGB3, ABCA4, KCNA2, ADRB2, SSTR2, SLC6A1, MCHR1, GRM1, MAS1, ADGRD1, PTPRC, SLCO4A1, SLCO2B1, CDHR1, FLRT1, GNRHR, PCDHA7.*
Molecular Function	GO:0042802; identical protein binding	18	0.00095	*GABRB3, SYT1, ITGB3, PRDM6, FOS, ADRB2, SLC6A1, MMP9, CXCL5, VIL1, ADAMTSL4, GIPC2, PTX3, ABCG4, PCDHA7, PVALB, MDFI, PFKP*
	GO:0005509; calcium ion binding	14	0.0011	*SYT1, PCDH10, VWDE, CACNA1E, VIL1, SCIN, EFEMP1, CDHR1, MGP, MYL10, PCDHA7, RPTN, PVALB, UMODL1.*

Supplementary Table 5. Gene Ontology enrichment analysis in Flight versus Ground old, sedentary derived myobundles

GO term categories	Term	Count	p-value	Genes
Cellular Component	GO:0005886; plasma membrane	93	7.86E-07	*CHRM2, CPM, IFITM1, CSF3R, GAL3ST1, SERPINE1, DIRAS2, SLC6A1, GLDN, GRM1, CDH8, RYR3, RASSF2, GJA5, SLCO2B1, TBC1D30, INSC, GSG1, GNAT1, NOS1, CA14, MINAR1, SERPINB2, ADGRV1, SCARA5, ACTN3, GPBAR1, RTN4RL2, RAB33A, ADRA2A, SLC9A3, ARC, NRN1L, ADGRB3, SLCO4A1, CDHR1, KCNQ1, MALL, PLIN4, AMPH, KCNA10, TRIB3, ADAM8, LCP1, PLCB2, FREM2, ABCG1, CD101, LLGL2, ITGA9, SERPINB12, KCNE5, ABCB4, NOXO1, ADRB2, SLC5A1, RASGRP2, CACNA1E, CORO1A, MCHR1, NKD1, DRP2, NKAIN4, RELN, PDZD2, NPBWR1, GALR1, CDH22, TM4SF20, PIP5K1B, TRPM8, SCN3A, KCNJ3, SLC38A4, SLC14A1, NGFR, MGAM, OR51E2, MX1, HSPA2, ADAP1, VIL1, MAS1, ADGRD1, KRT19, SLC6A9, P2RX1, KCNS3, CNTN3, PCDHB3, SCN2A, SDK2, CDK5R2.*
	GO:0016021; integral component of membrane	79	0.0039	*CHRM2, CNTNAP2, CPM, IFITM1, CSF3R, GAL3ST1, MYRFL, SLC6A1, CXCL13, GLDN, GRM1, CDH8, RYR3, GJA5, SLCO2B1, GSG1, CA14, BRICD5, MINAR1, ADGRV1, TMEM178B, GPBAR1, MID1, SLC9A3, CH25H, MMP13, ADGRB3, SLCO4A1, CDHR1, KCNQ1, PAPPA, TMEM217, MALL, UGT2B4, KCNA10, ADAM8, CTXN3, FREM2, ABCG1, CD101, TOR4A, OCA2, COL15A1, NDRG4, KCNE5, ABCB4, ILDR2, ADRB2, SLC5A1, MCHR1, KIAA0040, HSD11B1, HSD11B2, NKAIN4, NPBWR1, GALR1, CDH22, TM4SF20, GCNT3, TRPM8, SCN3A, CSMD1, KCNJ3, SLC38A4, SLC14A1, NGFR, MGAM, LINGO4, SLC37A2, CMTM5, OR51E2, DLK1, MAS1, ADGRD1, P2RX1, KCNS3, PCDHB3, SCN2A, SDK2.*
	GO:0016020; membrane	63	5.95E-04	*CHRM2, CNTNAP2, IFITM1, GAL3ST1, DIRAS2, STMN2, TFCP2L1, SLC6A1, RYR3, SERPINA5, SLCO2B1, GNAT1, CA14, BRICD5, ADGRV1, SLC6A17, TMEM178B, HBA2, CH25H, NRN1L, OAS1, ADGRB3, IRF4, CDHR1, KCNQ1, TMEM217, MALL, KCNA10, CTXN3, ABCG1, CD101, TOR4A, COL15A1, ABCB4, ILDR2, ADRB2, SLC5A1, CORO1A, KIAA0040, BEGAIN, HSD11B1, NPBWR1, GALR1, TM4SF20, GCNT3, CSMD1, IL32, NGFR, MGAM, LINGO4, CMTM5, MX1, SSC5D, HSPA2, DLK1, ADGRD1, SLC6A9, KCNS3, PCDHB3, SCN2A, CD24, PFKP, CDK5R2.*
	GO:0005576; extracellular region	45	0.000031	*TOR4A, DEFB103B, CPM, COL15A1, CSF3R, ECM2, PLGLB1, ELN, SERPINE1, PSG11, CXCL13, CXCL5, SERPINA5, SCUBE2, RELN, EFEMP1, PDZD2, ADAMTSL4, NTF3, PAMR1, RPTN, LGI1, CA11, NGFR, EGFL7, SERPINB2, SPAG6, HBA2, RTN4RL2, IGF1, LYZ, GDF6, MMP9, APLN, MFAP5, CHIT1, KISS1, MMP13, NRN1L, OAS1, PAPPA, ADM2, SAA1, MMP19, CNTN3.*

	**GO:0005615; extracellular space**	**43**	**1.61E-05**	***DEFB103B, CPM, COL15A1, SERPINB12, CPB1, CPXM2, SERPINE1, CXCL13, GLDN, CXCL5, SERPINA5, SCUBE2, RELN, EFEMP1, CHAD, NTF3, BRICD5, IL32, LGI1, LINGO4, EGFL7, SERPINB2, CMTM5, SSC5D, HBA2, RTN4RL2, IGF1, SERPINB7, C1QL4, LYZ, GDF6, MMP9, DLK1, APLN, CHIT1, KISS1, MMP13, KRT18, NRN1L, PAPPA, MMP19, LCP1, INA.***
	**GO:0005887; integral component of plasma membrane**	**30**	**0.001**	***CHRM2, CSF3R, GAL3ST1, ABCB4, ADRB2, SLC5A1, SLC6A1, MCHR1, GRM1, GJA5, NPBWR1, GALR1, SLCO2B1, TRPM8, SLC38A4, NGFR, SLC14A1, SCARA5, SLC6A17, ADRA2A, MAS1, ADGRD1, SLC6A9, ADGRB3, SLCO4A1, P2RX1, CDHR1, ADAM8, PCDHB3, SCN2A.***
	**GO:0009986; cell surface**	**16**	**0.0052**	***NGFR, CPM, CNTNAP2, EGFL7, ADGRV1, RTN4RL2, PSG11, HSPA2, SLC6A1, GLDN, SCUBE2, MAS1, SLC9A3, KCNQ1, ADAM8, CD24.***
**Molecular Function**	**GO:0005509; calcium ion binding**	**25**	**7.63E-06**	***RASGRP2, CACNA1E, NCALD, NKD1, CDH8, RYR3, SCUBE2, CAPN9, EFEMP1, CDH22, MYL10, PAMR1, RPTN, EGFL7, ADGRV1, ACTN3, DLK1, VIL1, MMP13, CDHR1, ADAM8, PCDHB3, LCP1, PLCB2, PVALB***
**Biological Process**	**GO:0007165; signal transduction**	**23**	**0.032**	***IL32, GUCY1A2, NDRG4, COL15A1, CSF3R, CMTM5, DIRAS2, MX1, IGF1, RASGRP2, ARHGAP36, PLCXD2, CXCL5, GLDN, APLN, SCUBE2, FAM83E, RASSF2, PPP1R1A, DPYSL5, P2RX1, NTF3, GNAT1.***
	**GO:0007155; cell adhesion**	**14**	**0.0115**	***IL32, CNTNAP2, COL15A1, EGFL7, CSF3R, CDH8, RELN, PDZD2, CDHR1, CNTN3, PCDHB3, CD24, FREM2, ITGA9.***

Supplementary Table 6. Differentially expressed genes in Ground old, sedentary vs Ground young, athletic derived myobundles

Gene	Description	Fold Change	FDR
	**Upregulated Genes (31)**		
ADH1B	All-trans-retinol dehydrogenase [NAD (+)]	5.50663	0.010542
AC093525.2	AC093525.2 readthrough	5.459093	0.009363
GRIA4	Glutamate receptor 4	5.317638	0.000103
SORCS1	VPS10 domain-containing receptor SorCS1	4.751788	5.84E-09
PGA4	Pepsin A-4	4.188893	5.66E-13
PGA3	Pepsin A-3	3.980967	8.90E-05
CD163	Scavenger receptor cysteine-rich type 1 protein M130	3.590494	0.001014
PGA5	Pepsin A-5	3.278504	1.29E-07
PCDHA10	Protocadherin alpha-10	3.194691	0.000562
CNTNAP3	Contactin-associated protein-like 3	2.952285	0.005689
PNLIPRP3	Pancreatic lipase-related protein 3	2.752608	0.002605
RASSF2	Ras association domain-containing protein 2	2.583977	0.001096
SEMA3E	Semaphorin-3E	2.503173	5.48E-05
EDIL3	EGF-like repeat and discoidin I-like domain-containing protein 3	2.478723	1.10E-19
SCUBE3	Signal peptide, CUB and EGF-like domain-containing protein 3	2.462808	0.040758
IFI44L	Interferon-induced protein 44-like	2.35222	0.000728
KCNJ4	Inward rectifier potassium channel 4	2.348139	0.002507
DOK6	Docking protein 6	2.323972	5.45E-05
GPRIN3	G protein-regulated inducer of neurite outgrowth 3	2.31117	0.013039
SNCA	Alpha-synuclein	2.306055	0.041873
BCHE	Cholinesterase	2.300941	0.01719
SCAMP5	Secretory carrier-associated membrane protein 5	2.270838	3.94E-13
CADM3	Cell adhesion molecule 3	2.23186	0.000649
MYMX	Protein myomixer	2.131104	0.002002
FAIM2	Protein lifeguard 2	2.122362	0.002104

GRIK2	Glutamate receptor ionotropic	2.122041	1.94E-15
ADORA2B	Adenosine receptor A2b	2.121165	2.30E-10
FAM155A	Transmembrane protein FAM155A	2.095744	0.000611
EPB41L4A	Band 4.1-like protein 4A	2.050197	0.033361
CPS1	Carbamoyl-phosphate synthase [ammonia], mitochondria	2.029799	4.82E-08
MAGEL2	MAGE-like protein 2	2.013006	0.039268
	**Downregulated Genes (46)**		
AC048338.1	AC048338.1 readthrough	-9.17064	0.000221
BARX1	Homeobox protein BarH-like 1	-7.57498	3.17E-06
CISH	Cytokine-inducible SH2-containing protein	-4.93738	0.001598
KIAA0040	Uncharacterized protein KIAA0040	-4.70838	2.70E-10
GAL3ST1	Galactosyl ceramide sulfotransferase	-4.4283	0.002806
SOCS3	Suppressor of cytokine signaling 3	-4.36672	0.000293
ST20-MTHFS	ST20-MTHFS readthrough	-4.29621	0.001366
STEAP4	Metalloreductase STEAP4	-4.17598	0.009271
TAC1	Protachykinin-1	-3.57829	8.90E-05
ABCC12	ATP-binding cassette sub-family C member 12	-3.42956	0.029524
EDN2	Endothelin-2	-3.39887	0.003263
CH25H	Cholesterol 25-hydroxylase	-3.38424	0.007555
GALNT6	Polypeptide N-acetylgalactosaminyltransferase 6	-3.34097	3.33E-05
SMTNL1	Smoothelin-like protein 1	-3.32285	2.64E-05
IL13RA2	Interleukin-13 receptor subunit alpha-2	-3.01697	0.001616
NOS2	Nitric oxide synthase, inducible	-2.93535	0.034761
CHRNA9	Neuronal acetylcholine receptor subunit alpha-9	-2.87794	0.000649
ALDH1L1	Cytosolic 10-formyltetrahydrofolate dehydrogenase	-2.87196	0.018974
ANO1	Anoctamin-1	-2.866	0.005077
RRAD	GTP-binding protein RAD	-2.82683	0.002793
RORC	Nuclear receptor ROR-gamma	-2.6904	1.46E-16
PDE4B	cAMP-specific 3',5'-cyclic phosphodiesterase 4B	-2.65437	0.000553
MB	Myoglobin	-2.64115	0.000845

CALML4	Calmodulin-like protein 4	-2.59142	1.31E-06
SEC14L5	SEC14-like protein 5	-2.57624	0.001385
NNAT	Neuronatin	-2.49611	1.01E-20
NKX2-3	Homeobox protein Nkx-2.3	-2.47777	0.028421
RPS10-NUDT3	RPS10-NUDT3 readthrough	-2.43414	5.92E-13
KCNRG	Potassium channel regulatory protein	-2.42893	0.017351
FAM153A	Protein FAM153A	-2.41254	0.015294
NGEF	Ephexin-1	-2.39547	0.002088
SLC9A3	Sodium/hydrogen exchanger 3	-2.25413	0.049253
ZFP36	mRNA decay activator protein ZFP36	-2.25365	5.48E-07
RGS16	Regulator of G-protein signaling 16	-2.20432	0.03053
GABRE	Gamma-aminobutyric acid receptor subunit epsilon	-2.20095	0.000936
PLCB2	1-phosphatidylinositol 4,5-bisphosphate phosphodiesterase beta-2	-2.19351	0.003512
CHL1	ATP-dependent DNA helicase DDX11	-2.16544	0.019322
TNXB	Tenascin-X	-2.15859	0.003185
SLX1A	Structure-Specific Endonuclease Subunit Homolog A,	-2.11698	0.003213
FOS	Proto-oncogene c-Fos	-2.11532	0.033171
CEBPD	CCAAT/enhancer-binding protein delta	-2.09462	9.32E-10
PLIN5	Perilipin-5	-2.0714	0.001877
GPR83	Probable G-protein coupled receptor 83	-2.0568	0.027382
CASQ1	Calsequestrin-1	-2.04761	0.000667
FOSB	FBJ murine osteosarcoma viral oncogene homolog B	-2.04146	0.010059
LLGL2	LLGL scribble cell polarity complex component 2	-2.00584	0.000611

Supplementary Table 7. Differentially expressed genes in Flight old, sedentary vs Flight young, athletic derived myobundles

Gene	Description	Fold Change	FDR
	**Upregulated Genes (44)**		
ADH1B	All-trans-retinol dehydrogenase [NAD(+)] ADH1B	6.476466606	0.004267711
SULT1A4	Sulfotransferase 1A4	6.46519923	0.009108532
PCDHA10	Protocadherin alpha-10	5.683026627	8.92125E-06
OAS1	2'-5'-oligoadenylate synthase 1	5.15144673	0.000673293
SORCS1	VPS10 domain-containing receptor SorCS1	4.481635518	1.21033E-11
CBSL	Cystathionine beta-synthase-like protein	4.441135365	0.018931424
MX1	Interferon-induced GTP-binding protein Mx1	4.149613978	1.13863E-07
CD163	Scavenger receptor cysteine-rich type 1 protein M130	3.737039114	0.002153255
MMP9	Matrix metalloproteinase-9	3.734812778	0.013499832
TMEM110-MUSTN1	TMEM110-MUSTN1 readthrough	3.706558203	0.000207655
COL28A1	Collagen alpha-1(XXVIII) chain	3.701382513	0.002712071
SYT1	Synaptotagmin-1	3.689683161	0.001941688
PGA3	Pepsin A-3	3.543055223	0.000678393
IFI44L	Interferon-induced protein 44-like	3.409816459	8.1528E-08
OAS3	2'-5'-oligoadenylate synthase 3	3.375568637	1.84192E-09
CADM3	Cell adhesion molecule 3	3.358524934	4.93045E-07
PGA4	Pepsin A-4	3.349734128	2.92566E-08
CNTNAP3	Contactin-associated protein-like 3	3.330953921	0.000212932
ANO2	Anoctamin-2	3.330086633	0.015099819
TFCP2L1	Transcription factor CP2-like protein 1	3.313716199	0.016620049
IFI27	Interferon alpha-inducible protein 27, mitochondrial	3.29935305	0.011949739
NOV	CCN family member 3	3.293005515	0.000473995
IFITM1	Interferon-induced transmembrane protein 1	3.222678754	1.84028E-05
EPSTI1	Epithelial-stromal interaction protein 1	3.148236733	0.008146499
SNCA	Alpha-synuclein	3.08909594	0.012642524
SFRP5	Secreted frizzled-related protein 5	3.008324388	0.012206691

SFRP1	Secreted frizzled-related protein 1	2.963010715	0.002429386
MYMX	Protein myomixer	2.841541397	9.32065E-06
EPHA3	Ephrin type-A receptor 3	2.770209347	0.002479832
GABRB3	Gamma-aminobutyric acid receptor subunit beta-3	2.739611434	0.000818999
PLA2G4A	Cytosolic phospholipase A2	2.641157626	0.005201275
EIF2S3B	Eukaryotic translation initiation factor 2 subunit 3B	2.465893665	0.000828289
FAM9C	Family with sequence similarity 9 member C	2.436032937	0.001740291
RIMS3	Regulating synaptic membrane exocytosis protein 3	2.432061242	0.001804696
ADORA2B	Adenosine receptor A2b	2.398180367	4.22786E-11
PWP2	Periodic tryptophan protein 2 homolog	2.36903012	0.000155964
TP53TG3D		2.345348958	0.042210595
PGA5	Pepsin A-5	2.330090118	0.000579969
FHAD1	Forkhead-associated domain-containing protein 1	2.311771577	0.031987124
EDIL3	EGF-like repeat and discoidin I-like domain-containing protein 3	2.305432437	1.21792E-15
SLC13A4	Solute carrier family 13 member 4	2.045267982	0.027502872
IFIH1	Interferon-induced helicase C domain-containing protein 1	2.028135888	0.000847821
SIK1	Serine/threonine-protein kinase SIK1	2.018609418	8.60471E-16
ACSS3	Acyl-CoA synthetase short-chain family member 3, mitochondrial	2.010982813	3.72392E-07
	**Downregulated Genes (22)**		
NNAT	Neuronatin	-2.25377	8.6E-16
MAPK4	Mitogen-activated protein kinase 4	-2.38982	9.47E-10
MYH2	Myosin heavy chain 2	-2.14487	2.2E-08
STAC2	SH3 and cysteine-rich domain-containing protein 2	-2.13934	4.74E-08
OSTN	Osteocrin	-2.03364	8.09E-08
ACAN	Aggrecan core protein	-3.00922	1.55E-06
ZNF85	Zinc finger protein 85	-2.1116	5.89E-05
ARHGEF33	Rho guanine nucleotide exchange factor 33	-3.03639	6.74E-05
ACTN3	Alpha-actinin-3	-2.18816	0.000178
BARX1	Homeobox protein BarH-like 1	-6.23299	0.000242

TSHZ2	Teashirt homolog 2	-2.25819	0.000374
PDZRN4	PDZ domain-containing RING finger protein 4	-2.34787	0.002614
WISP1	CCN family member 4	-2.91471	0.004592
TMEM154	Transmembrane protein 154	-2.33647	0.007467
ZNF781	Zinc finger protein 781	-3.92229	0.008469
ADGRB3	Adhesion G protein-coupled receptor B3	-4.23641	0.010137
COMP	Cartilage oligomeric matrix protein	-3.95436	0.012927
HIST1H1E	H1.4 linker histone	-3.91815	0.013631
TRPV3	Transient receptor potential cation channel subfamily V member 3	-2.29342	0.021744
GALNT6	Polypeptide N-acetylgalactosaminyltransferase 6	-2.05224	0.021867
B3GNT4	N-acetyllactosaminide beta-1,3-N-acetylglucosaminyltransferase 4	-2.43406	0.027832
ABCC3	ATP-binding cassette sub-family C member 3	-2.00106	0.041299

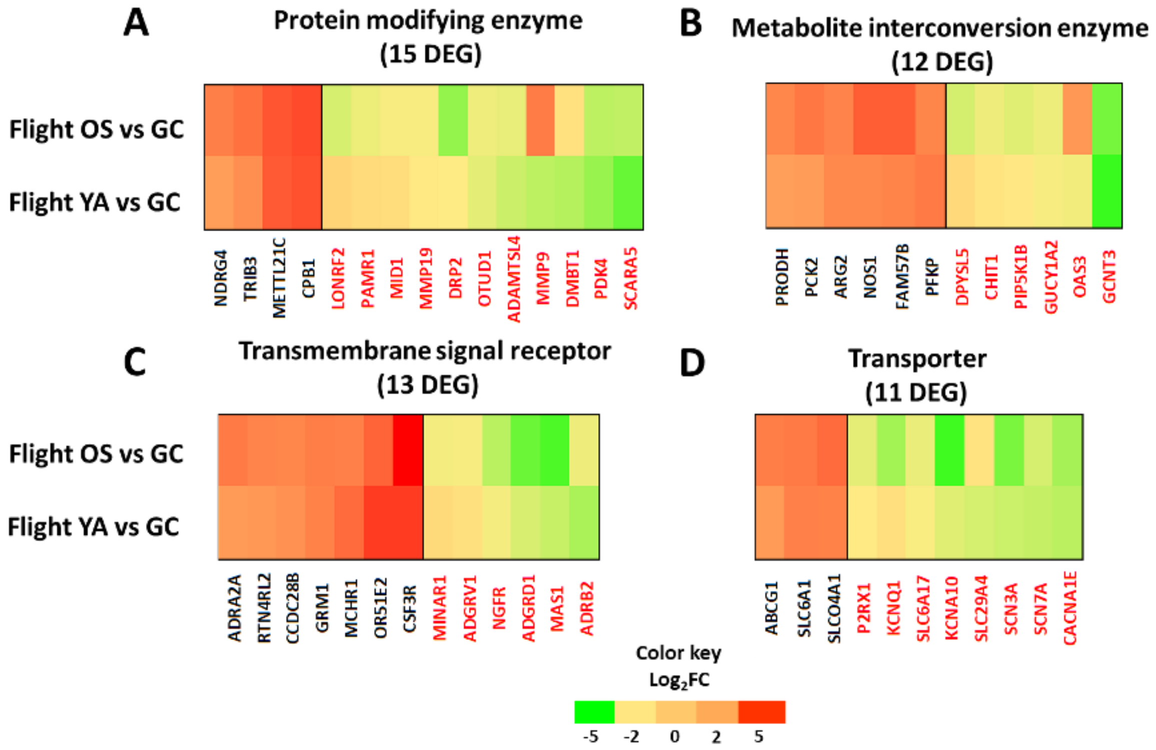

Supplementary Figure 1. **Panther classification system analysis**. Comparative heat map gene expression of the overlapping DEGs among the mean YA and mean OS flight versus ground datasets. DEGs associated with microgravity were normalized to GC and color coded as log2FC from -5 to 5, with a p-value ≤0.05. The log2 fold change (log2FC) values for the involved genes were extracted and displayed as heatmaps. Each chip is derived from cells pooled from 5 donors of the YA cohort or the OS cohort. Overrepresentation is shown as a significant fold enrichment based on the PANTHER overrepresentation test using Fisher's exact test with FDR multiple correction ($P \leq 0.05$) and *Homo Sapiens* as reference organism. Three chips from each cohort are used in the analysis.

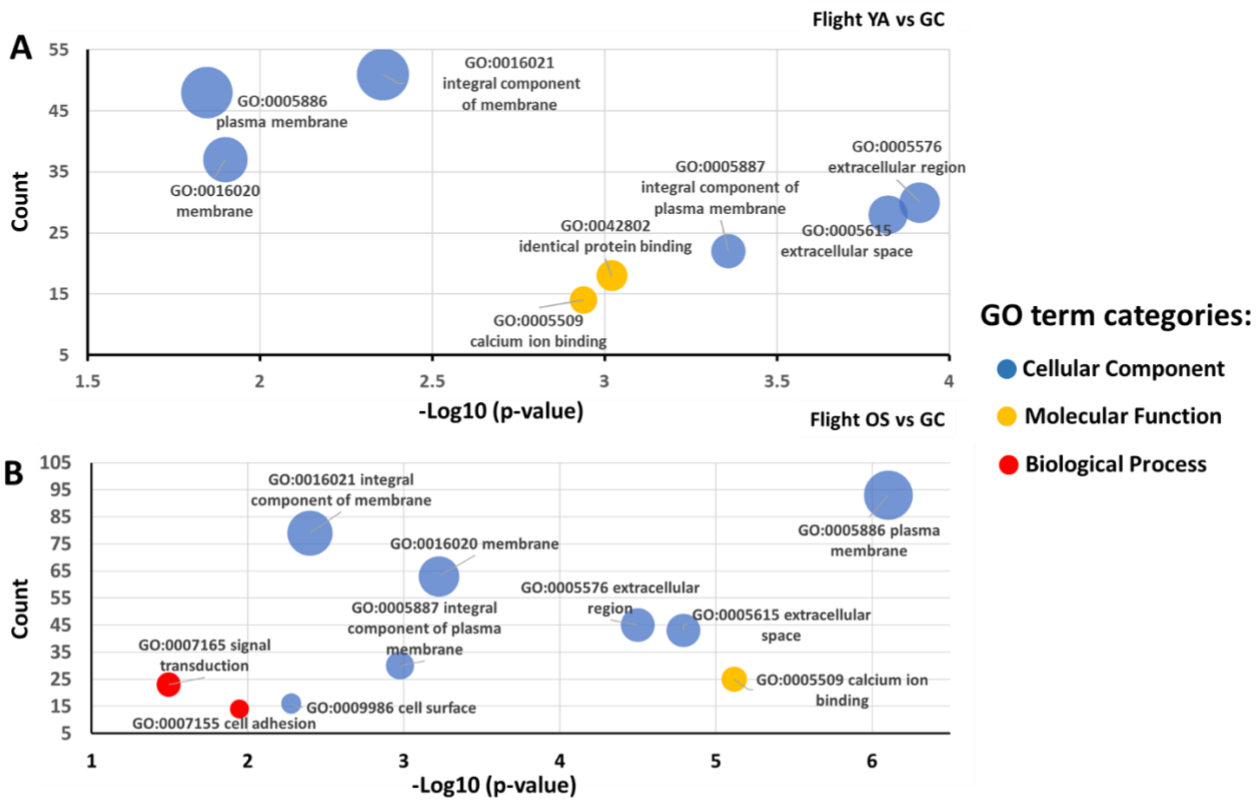

Supplementary Figure 2. **Gene ontology (GO) annotation**. Bubble plots of differentially expressed gene sets overrepresented in the DAVID GO database. The analysis was performed separately for each experimental group. **A**. Flight YA vs. GC and **B**. Flight OS vs. GC. The graphs show only the GO terms above the established cut-off criteria (p-values ≤ 0.05, $|\log FC| \geq 2$ and ≥ 14 genes per group). The x-axis represents the negative logarithm of the adjusted p value [false discovery rate (FDR)] for the GO terms, and the y-axis displays count, so each bubble's size reflects the number of differentially expressed genes, assigned to the GO terms. The top terms in each category, cellular component, molecular function, and biological process, are displayed in blue, orange, and red, respectively. The enriched functional GO terms associated with our gene list are identified and listed according to their enrichment P-value by DAVID using Fisher's Exact p-values test.

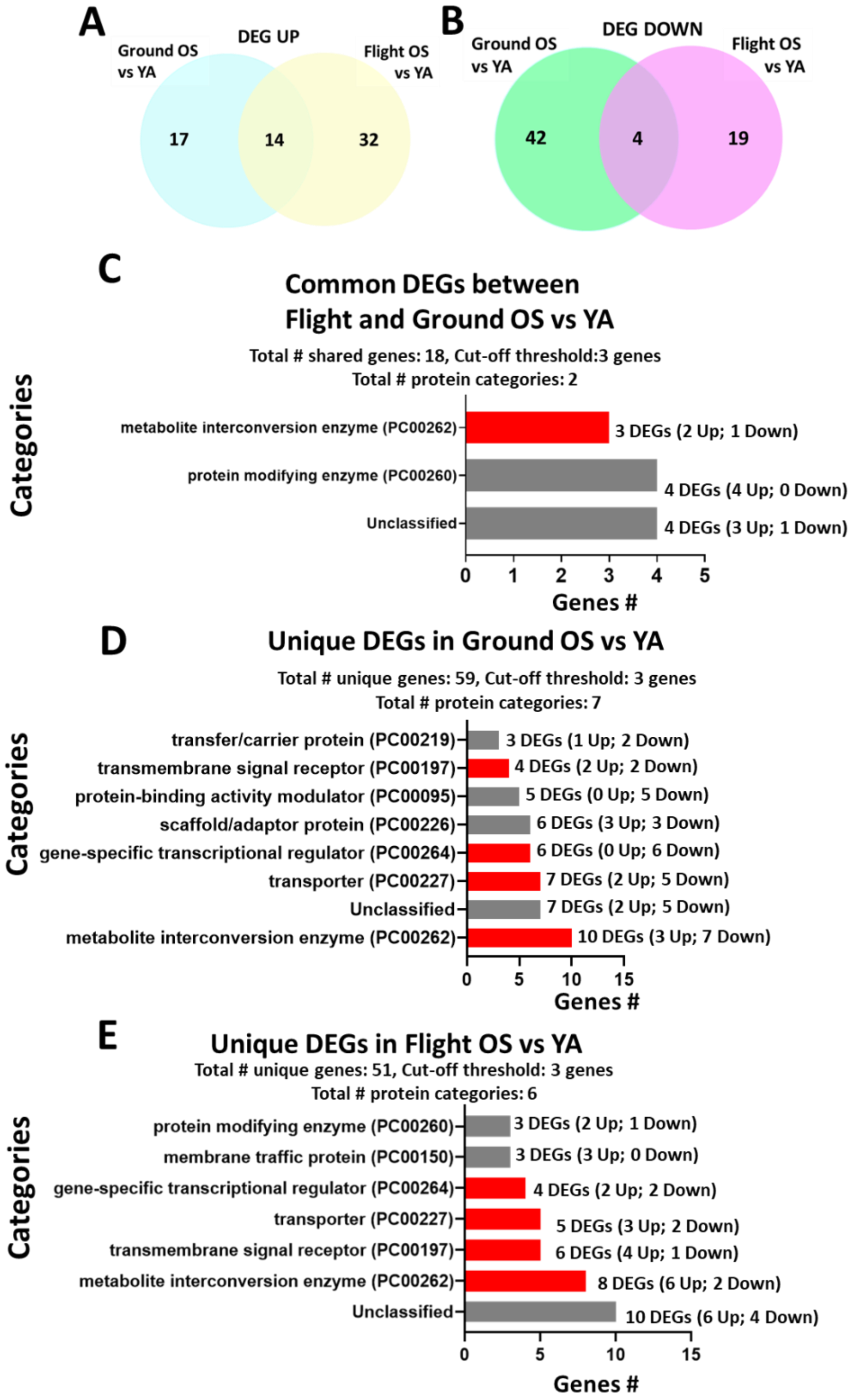
A
DEG UP
Ground OS vs YA
Flight OS vs YA
17
14
32
B
DEG DOWN
Ground OS vs YA
Flight OS vs YA
42
4
19
C
Common DEGs between Flight and Ground OS vs YA
Total # shared genes: 18, Cut-off threshold:3 genes
Total # protein categories: 2
Categories
metabolite interconversion enzyme (PC00262)
3 DEGs (2 Up; 1 Down)
protein modifying enzyme (PC00260)
4 DEGs (4 Up; 0 Down)
Unclassified
4 DEGs (3 Up; 1 Down)
0 1 2 3 4 5
Genes #
D
Unique DEGs in Ground OS vs YA
Total # unique genes: 59, Cut-off threshold: 3 genes
Total # protein categories: 7
Categories
transfer/carrier protein (PC00219)
3 DEGs (1 Up; 2 Down)
transmembrane signal receptor (PC00197)
4 DEGs (2 Up; 2 Down)
protein-binding activity modulator (PC00095)
5 DEGs (0 Up; 5 Down)
scaffold/adaptor protein (PC00226)
6 DEGs (3 Up; 3 Down)
gene-specific transcriptional regulator (PC00264)
6 DEGs (0 Up; 6 Down)
transporter (PC00227)
7 DEGs (2 Up; 5 Down)
Unclassified
7 DEGs (2 Up; 5 Down)
metabolite interconversion enzyme (PC00262)
10 DEGs (3 Up; 7 Down)
0 5 10 15
Genes #
E
Unique DEGs in Flight OS vs YA
Total # unique genes: 51, Cut-off threshold: 3 genes
Total # protein categories: 6
Categories
protein modifying enzyme (PC00260)
3 DEGs (2 Up; 1 Down)
membrane traffic protein (PC00150)
3 DEGs (3 Up; 0 Down)
gene-specific transcriptional regulator (PC00264)
4 DEGs (2 Up; 2 Down)
transporter (PC00227)
5 DEGs (3 Up; 2 Down)
transmembrane signal receptor (PC00197)
6 DEGs (4 Up; 1 Down)
metabolite interconversion enzyme (PC00262)
8 DEGs (6 Up; 2 Down)
Unclassified
10 DEGs (6 Up; 4 Down)
0 5 10 15
Genes #

Supplementary Figure 3. Unique and common DEGs comparing ground OS vs YA and flight OS vs YA datasets. a. b. Venn diagrams showing the number of up-regulated and down-regulated DEGs identified by RNA-Seq among the two comparisons. DEGs only expressed in the ground comparison (light blue and green circles), only expressed in the flight comparison (yellow and circles), and common to both comparisons (intersections). c. d. e. Classification by Panther classification system of shared and unique DEGs shown in the Venn diagrams. The horizontal bar chart identifies the protein classes (unique gray, shared red) and relative number of DEGs of each functional class by applying a cut-off threshold of 3 genes. The protein categories are entitled with protein classifiers (PC) codes. Overrepresentation is shown as a significant fold enrichment based on the PANTHER overrepresentation test using Fisher's exact test with FDR multiple correction ($P \leq 0.05$) and *Homo Sapiens* as reference organism.
