# Supplementary material for: Human skeletal muscle tissue chip autonomous payload reveals changes in fiber type and metabolic gene expression due to spaceflight
Source: NPJ Microgravity. 2023 Sep 15;9:77. doi: 10.1038/s41526-023-00322-y (PMC10504373; doi:10.1038/s41526-023-00322-y)
Supplement: Supplementary file 2 — Reporting Summary [file 41526_2023_322_MOESM2_ESM.pdf]

nature portfolio

Corresponding author(s): Siobhan Malany

Last updated by author(s): Jun 07 2023

Reporting Summary

Nature Portfolio wishes to improve the reproducibility of the work that we publish. This form provides structure for consistency and transparency in reporting. For further information on Nature Portfolio policies, see our Editorial Policies and the Editorial Policy Checklist.

Statistics

For all statistical analyses, confirm that the following items are present in the figure legend, table legend, main text, or Methods section.

n/a	Confirmed	
☐	☒	The exact sample size (*n*) for each experimental group/condition, given as a discrete number and unit of measurement
☐	☒	A statement on whether measurements were taken from distinct samples or whether the same sample was measured repeatedly
☒	☐	The statistical test(s) used AND whether they are one- or two-sided<br>*Only common tests should be described solely by name; describe more complex techniques in the Methods section.*
☐	☒	A description of all covariates tested
☒	☐	A description of any assumptions or corrections, such as tests of normality and adjustment for multiple comparisons
☐	☒	A full description of the statistical parameters including central tendency (e.g. means) or other basic estimates (e.g. regression coefficient) AND variation (e.g. standard deviation) or associated estimates of uncertainty (e.g. confidence intervals)
☐	☒	For null hypothesis testing, the test statistic (e.g. *F*, *t*, *r*) with confidence intervals, effect sizes, degrees of freedom and *P* value noted<br>*Give P values as exact values whenever suitable.*
☒	☐	For Bayesian analysis, information on the choice of priors and Markov chain Monte Carlo settings
☒	☐	For hierarchical and complex designs, identification of the appropriate level for tests and full reporting of outcomes
☒	☐	Estimates of effect sizes (e.g. Cohen's *d*, Pearson's *r*), indicating how they were calculated

Our web collection on statistics for biologists contains articles on many of the points above.

Software and code

Policy information about availability of computer code

Data collection	Quality control was performed before and after trimming using FastQC (v 0.11.4) and MultiQC53. Transcript abundance was quantified using RSEM (RSEM v1.3.1). Differential expression analysis was performed using the DESeq2 package
Data analysis	iPathwayGuide (Advaita Bioinformatics http://www.advaitabio.com/ipathwayguide.html), Database for Annotation, Visualization, and Integrated Discovery database (DAVID, https://david.ncifcrf.gov/), and Protein Analysis Through Evolutionary Relationships (PANTHER v.13.0, www.pantherdb.org)

For manuscripts utilizing custom algorithms or software that are central to the research but not yet described in published literature, software must be made available to editors and reviewers. We strongly encourage code deposition in a community repository (e.g. GitHub). See the Nature Portfolio guidelines for submitting code & software for further information.

Data

Policy information about availability of data

All manuscripts must include a data availability statement. This statement should provide the following information, where applicable:

- Accession codes, unique identifiers, or web links for publicly available datasets
- A description of any restrictions on data availability
- For clinical datasets or third party data, please ensure that the statement adheres to our policy

All data generated during this study are either included in the manuscript and its Supplementary files or are available from the corresponding authors upon

reasonable request. RNASeq data are available at the Gene Expression Omnibus (GEO) database (XXX). Patient biopsy cells were obtained from AdventHealth Orlando through a Material Transfer Agreement to the University of Florida with restrictions for sharing with a third party.

Research involving human participants, their data, or biological material

Policy information about studies with human participants or human data. See also policy information about sex, gender (identity/presentation), and sexual orientation and race, ethnicity and racism.

Reporting on sex and gender	Sex is a biological variable in aging muscle. Because the sample size was limited by the size of the 9U Cubelab, we chose to include only cells from male donors in this experiment such that we could include both young and older cohorts.
Reporting on race, ethnicity, or other socially relevant groupings	To investigate the differences in bio engineered muscle in flight versus ground, we used cells from white, male donors to best provide reproducible data and limit variances due to differences in race and ethnicity of donors. The objective of the study is to provide a foundation to standardize and adapt these microphysiological systems integrated into autonomous CubeLab platforms for space use.
Population characteristics	AdventHealth, Translational Research Institute (TRI) obtained percutaneous biopsies of the vastus lateralis from five young, athletic men (<40 yrs, YA) and five older, sedentary men (>60 yrs, OS) volunteers. A participant was considered athletic or sedentary based on their level of routine endurance exercise (running, cycling, or swimming) of at least 3 days a week or one day or less a week, respectively. UF researchers recieved muscle precursor cells from these samples through a Material Transfer Agreement.
Recruitment	AdventHealth recruited participants and all participants provided written informed consent
Ethics oversight	the study protocol was reviewed and approved by the institutional review board at AdventHealth, Orlando (IRBNet #554559). All data provided to Dr. Malany and her staff at UF is de-identified data. At AVH-TRI, all study data was collected by the research team, reviewed by Dr. Coen, and stored in secure, locked files and/or password protected electronic databases in the immediate offices and laboratories of the research team to protect from inadvertent loss or improper access

Note that full information on the approval of the study protocol must also be provided in the manuscript.

Field-specific reporting

Please select the one below that is the best fit for your research. If you are not sure, read the appropriate sections before making your selection.

☒ Life sciences ☐ Behavioural & social sciences ☐ Ecological, evolutionary & environmental sciences

For a reference copy of the document with all sections, see nature.com/documents/nr-reporting-summary-flat.pdf

Life sciences study design

All studies must disclose on these points even when the disclosure is negative.

Sample size	Three distinct tissue chips were used in each group for RNA-Seq analysis to establish significance and p-value determination
Data exclusions	We excluded the tissue chips that were electrically stimulated one time because there was no significant difference in differential genes between stimulated or non-stimulated tissue chips. We choose three of four tissue chips tested from each cohort for RNA-Seq analysis for budgetary reasons.
Replication	Cells from four donors were pooled in equal ratios and aliquoted to ensure the same stock of mean cells were used for flight and ground studies. triplicate tissue chips were used in the RNA-Seq analysis
Randomization	we obtained percutaneous biopsies of the vastus lateralis from five young, athletic men (<40 yrs, YA) and five older, sedentary men (>60 yrs, OS) volunteers. A participant was considered athletic or sedentary based on their level of routine endurance exercise (running, cycling, or swimming) of at least 3 days a week or one day or less a week, respectively. Since gender differences represent a biological variable in aging, we choose to use cells from only men for this flight and ground experiment.
Blinding	Data collection from flight studies was recorded by the CubeLab and downloaded into the Space Tango portal. Secure access to the portal was provided to UF investigators. RNA-Seq Data analysis was performed by the Interdisciplinary Center for Biotechnology Research at UF and data was provided to UF investigators.

Reporting for specific materials, systems and methods

We require information from authors about some types of materials, experimental systems and methods used in many studies. Here, indicate whether each material, system or method listed is relevant to your study. If you are not sure if a list item applies to your research, read the appropriate section before selecting a response.

Materials & experimental systems

n/a	Involved in the study	
☒	☐	Antibodies
☒	☐	Eukaryotic cell lines
☒	☐	Palaeontology and archaeology
☒	☐	Animals and other organisms
☒	☐	Clinical data
☒	☐	Dual use research of concern
☒	☐	Plants

Methods

n/a	Involved in the study	
☒	☐	ChIP-seq
☒	☐	Flow cytometry
☒	☐	MRI-based neuroimaging
